# Effectiveness of Home-Based Mobile Guided Cardiac Rehabilitation as Alternative Strategy for Nonparticipation in Clinic-Based Cardiac Rehabilitation Among Elderly Patients in Europe: A Randomized Clinical Trial

**DOI:** 10.1001/jamacardio.2020.5218

**Published:** 2020-10-28

**Authors:** Johan A. Snoek, Eva I. Prescott, Astrid E. van der Velde, Thijs M. H. Eijsvogels, Nicolai Mikkelsen, Leonie F. Prins, Wendy Bruins, Esther Meindersma, José R. González-Juanatey, Carlos Peña-Gil, Violeta González-Salvado, Feriel Moatemri, Marie-Christine Iliou, Thimo Marcin, Prisca Eser, Matthias Wilhelm, Arnoud W.J. Van’t Hof, Ed P. de Kluiver

**Affiliations:** 1 Isala Heart Center, Zwolle, the Netherlands; 2 Sports Medicine Department Isala, Zwolle, the Netherlands; 3 Department of Cardiology, Bispebjerg Frederiksberg University Hospital, Copenhagen, Denmark; 4 Radboud Institute for Health Sciences, Department of Physiology, Radboud University Medical Center, Nijmegen, the Netherlands; 5 Diagram, Zwolle, the Netherlands; 6 Department of Cardiology, Radboud University Medical Center, Nijmegen, the Netherlands; 7 Department of Cardiology, Hospital Clínico Universitario de Santiago, Instituto de Investigación Sanitaria, CIBER CV, Madrid, Spain; 8 Department of Cardiac Rehabilitation, Assistance Publique Hopitaux de Paris, Paris, France; 9 Department of Cardiology, Inselspital, Bern University Hospital, University of Bern, Bern, Switzerland; 10 Department of Cardiology, Maastricht University Medical Center and Cardiovascular Research Institute Maastricht (CARIM), Maastricht, the Netherlands; 11 Department of Cardiology, Zuyderland Medical Center, Heerlen, the Netherlands

## Abstract

**Importance:**

Although nonparticipation in cardiac rehabilitation is known to increase cardiovascular mortality and hospital readmissions, more than half of patients with coronary artery disease in Europe are not participating in cardiac rehabilitation.

**Objective:**

To assess whether a 6-month guided mobile cardiac rehabilitation (MCR) program is an effective therapy for elderly patients who decline participation in cardiac rehabilitation.

**Design, Setting, and Participants:**

Patients were enrolled in this parallel multicenter randomized clinical trial from November 11, 2015, to January 3, 2018, and follow-up was completed on January 17, 2019, in a secondary care system with 6 cardiac institutions across 5 European countries. Researchers assessing primary outcome were masked for group assignment. A total of 4236 patients were identified with a recent diagnosis of acute coronary syndrome, coronary revascularization, or surgical or percutaneous treatment for valvular disease, or documented coronary artery disease, of whom 996 declined to start cardiac rehabilitation. Subsequently, 179 patients who met the inclusion and exclusion criteria consented to participate in the European Study on Effectiveness and Sustainability of Current Cardiac Rehabilitation Programmes in the Elderly trial. Data were analyzed from January 21 to October 11, 2019.

**Interventions:**

Six months of home-based cardiac rehabilitation with telemonitoring and coaching based on motivational interviewing was used to stimulate patients to reach exercise goals. Control patients did not receive any form of cardiac rehabilitation throughout the study period.

**Main Outcomes and Measures:**

The primary outcome parameter was peak oxygen uptake (Vo_2_peak) after 6 months.

**Results:**

Among 179 patients randomized (145 male [81%]; median age, 72 [range, 65-87] years), 159 (89%) were eligible for primary end point analysis. Follow-up at 1 year was completed for 151 patients (84%). Peak oxygen uptake improved in the MCR group (n = 89) at 6 and 12 months (1.6 [95% CI, 0.9-2.4] mL/kg^−1^/min^−1^ and 1.2 [95% CI, 0.4-2.0] mL/kg^−1^/min^−1^, respectively), whereas there was no improvement in the control group (n = 90) (+0.2 [95% CI, −0.4 to 0.8] mL/kg^−1^/min^−1^ and +0.1 [95% CI, −0.5 to 0.7] mL/kg^−1^/min^−1^, respectively). Changes in Vo_2_peak were greater in the MCR vs control groups at 6 months (+1.2 [95% CI, 0.2 to 2.1] mL/kg^−1^/min^−1^) and 12 months (+0.9 [95% CI, 0.05 to 1.8] mL/kg^−1^/min^−1^). The incidence of adverse events was low and did not differ between the MCR and control groups.

**Conclusions and Relevance:**

These results suggest that a 6-month home-based MCR program for patients 65 years or older with coronary artery disease or a valvular intervention was safe and beneficial in improving Vo_2_peak when compared with no cardiac rehabilitation.

**Trial Registration:**

trialregister.nl Identifier: NL5168

## Key Points

**Question:**

Is home-based cardiac rehabilitation an effective therapy for elderly patients who decline conventional cardiac rehabilitation?

**Findings:**

In this international, multicenter, randomized clinical trial including 179 patients, 6 months of home-based mobile cardiac rehabilitation was associated with a greater increase in physical fitness compared with no cardiac rehabilitation. These beneficial adaptations were sustainable at 1-year follow-up, whereas the incidence of adverse events was low and similar between the intervention and control groups.

**Meaning:**

These findings suggest that home-based mobile cardiac rehabilitation for elderly patients is safe and effective in changing physical fitness for patients that are not able or willing to participate in conventional cardiac rehabilitation.

## Teaser

This randomized clinical study assesses whether a 6-month mobile cardiac rehabilitation program is an effective therapy for elderly patients who decline participation in cardiac rehabilitation.

## Introduction

Approximately 50% of patients with coronary artery disease are not referred to participate in cardiac rehabilitation.^1^ This statistic is even worse for elderly patients, despite their higher prevalence of comorbidities and a less physically active lifestyle.^2^ Transportation difficulties are a key reason for nonparticipation and further contribute to the lower cardiac rehabilitation participation rates of older patients.^3^ Home-based mobile cardiac rehabilitation (MCR) programs have been suggested as an alternative for the elderly patient unable or unwilling to participate in center-based cardiac rehabilitation.^4^

The aim of the present study was to assess whether MCR is an effective therapy for patients 65 years or older who decline participation in a conventional cardiac rehabilitation program. We hypothesized that MCR would lead to (1) better physical fitness at 6-month follow-up, (2) a sustained effect at 12-month follow-up, (3) an increase in habitual physical activity, (4) improved cardiovascular risk factors, and (5) lower incidence of adverse events.

## Methods

Details of the study methods of the European Study on Effectiveness and Sustainability of Current Cardiac Rehabilitation Programmes in the Elderly (EU-CaRE) randomized clinical trial (RCT) have been published previously^5^; a copy of the trial protocol is available in Supplement 1. Further details are given in the eMethods in Supplement 2. In short, this multicenter RCT was conducted in accordance with the Declaration of Helsinki.^6^ Six cardiac institutions across 5 European countries participated. The study protocol was approved by all local ethics committees, and informed written consent was obtained from all participants. This study followed Consolidated Standards of Reporting Trials (CONSORT) reporting guideline.

Patients 65 years or older with a recent diagnosis (<3 months) of acute coronary syndrome, coronary revascularization, surgical or percutaneous treatment for valvular disease, or documented coronary artery disease defined by standard noninvasive or invasive methods and who declined participation in center-based cardiac rehabilitation were eligible for inclusion. Patients were recruited by a research assistant from the participating centers by screening all consecutive patients admitted to the participating centers. Randomization was performed in fixed blocks of 4, stratified by center, with a 1:1 ratio to the intervention group (MCR) or a control group without cardiac rehabilitation using a centralized computerized allocation system. Researchers assessing primary outcomes were blinded for group assignment.

Patients in the MCR group were offered a 6-month home-based cardiac rehabilitation program in which they were equipped with a smartphone and heart rate belt. Patients were instructed to exercise at moderate intensity for at least 30 minutes per day, 5 days per week.^7^ Motivational interviewing was applied by telephone: weekly in the first month, every other week in the second month, and monthly until completion of the MCR program at 6 months (eFigure 1 in Supplement 2). After 6 months, the home-based cardiac rehabilitation program was finished and equipment was handed in. Patients in the control group did not receive any form of cardiac rehabilitation but received locally defined standard of care.

The primary end point was the difference in change in physical fitness from baseline to 6-month follow-up between MCR and controls. Fitness was defined as the highest 30-second moving mean peak of oxygen uptake (Vo_2_peak). Self-reported physical activity was assessed using the following 2 questions: “How many days per week do you perform moderate to vigorous PA [physical activity]?” and “How many minutes per day do you perform moderate to vigorous PA?” The definition of self-reported habitual physical activity was considered the total number of days per week in which a minimum of 30 minutes of self-reported moderate to vigorous physical activity was registered. Standardized clinical chemical blood tests were performed to determine levels of total cholesterol, low-density and high-density lipoprotein cholesterol, and hemoglobin A_1c_ (HbA_1c_) in local laboratories. Adverse events were registered and collected by monthly telephone calls with participants in both groups and evaluation of patients’ electronic medical files. A Clinical Event Committee reviewed and adjudicated all clinical end point events.

Data were analyzed from January 21 to October 11, 2019. All parameters were analyzed according to the initial randomization. Analyses were performed using SAS, version 9.4 (SAS Institute, Inc). The primary outcome was assessed by a linear mixed model with change in Vo_2_peak (6 months difference from baseline) as response, the intervention (MCR vs control) as a fixed effect, the effect of center as a random effect, and adjustment for baseline value of Vo_2_peak. Two-sided *P* < .05 was considered statistically significant.

## Results

**Figure 1.**
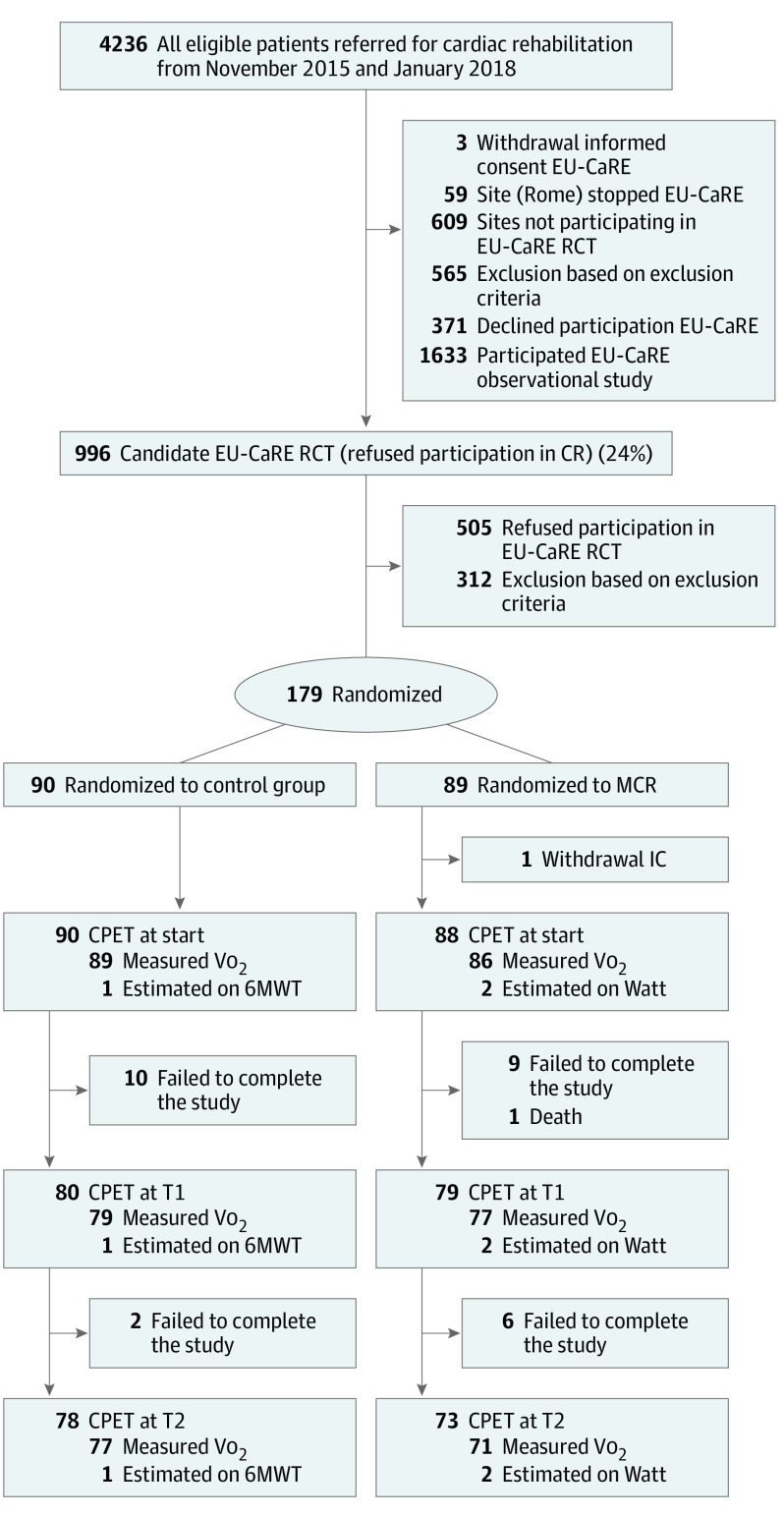
Flowchart (CONSORT) of All Eligible Patients Referred for Cardiac Rehabilitation in Centers Screened for the European Study on Effectiveness and Sustainability of Current Cardiac Rehabilitation Programmes in the Elderly (EU-CaRE) A total of 996 patients (24%) declined participation in cardiac rehabilitation, of whom 312 were excluded from the EU-CaRE randomized clinical trial. Of the remaining 684 patients, a total of 179 were willing to participate (26%). CPET indicates cardiopulmonary exercise test; MCR, mobile cardiac rehabilitation; Vo_2_, oxygen uptake; and 6MWT 6-minute walking test.

From November 11, 2015, to January 3, 2018, a total of 179 of 684 eligible patients (26%) who declined participation in center-based cardiac rehabilitation were included in the study. A CONSORT flowchart of the trial is presented in Figure 1. The primary reason for nonparticipation was a perceived lack of usefulness (eFigure 2 in Supplement 2). The trial (including follow-up) ended January 17, 2019, owing to reaching the end date set by the funder. The median age of participants was 72 (range, 65-87) years, with 20 patients (11%) older than 80 years (eTable 1 in Supplement 2). Thirty-four patients were female (19%) and 145 were male (81%). No differences were found in patient characteristics, index event for cardiac rehabilitation, medication use, and baseline Vo_2_peak. Patients in the MCR group were more often diagnosed with hypertension (73 of 89 [82%] vs 60 of 90 [67%]).

**Figure 2.**
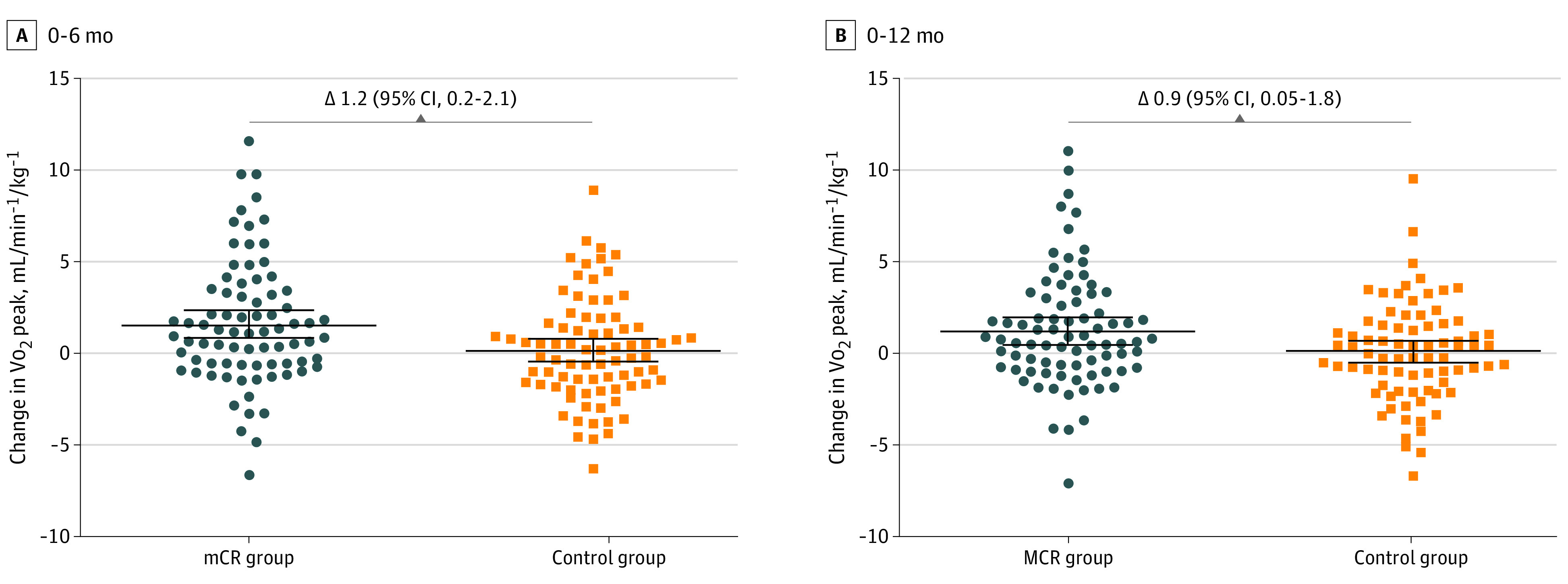
Primary Outcome of Change in Peak Oxygen Uptake (Vo_2_peak) at 6 and 12 Months of Follow-up Change in Vo_2_peak from baseline to 6 and 12 months follow-up is significantly greater in the mobile cardiac rehabilitation (MCR) intervention group compared with the control group. Data are presented as mean (95% CI). The difference between groups with 95% CI is displayed at the top.

**Figure 3.**
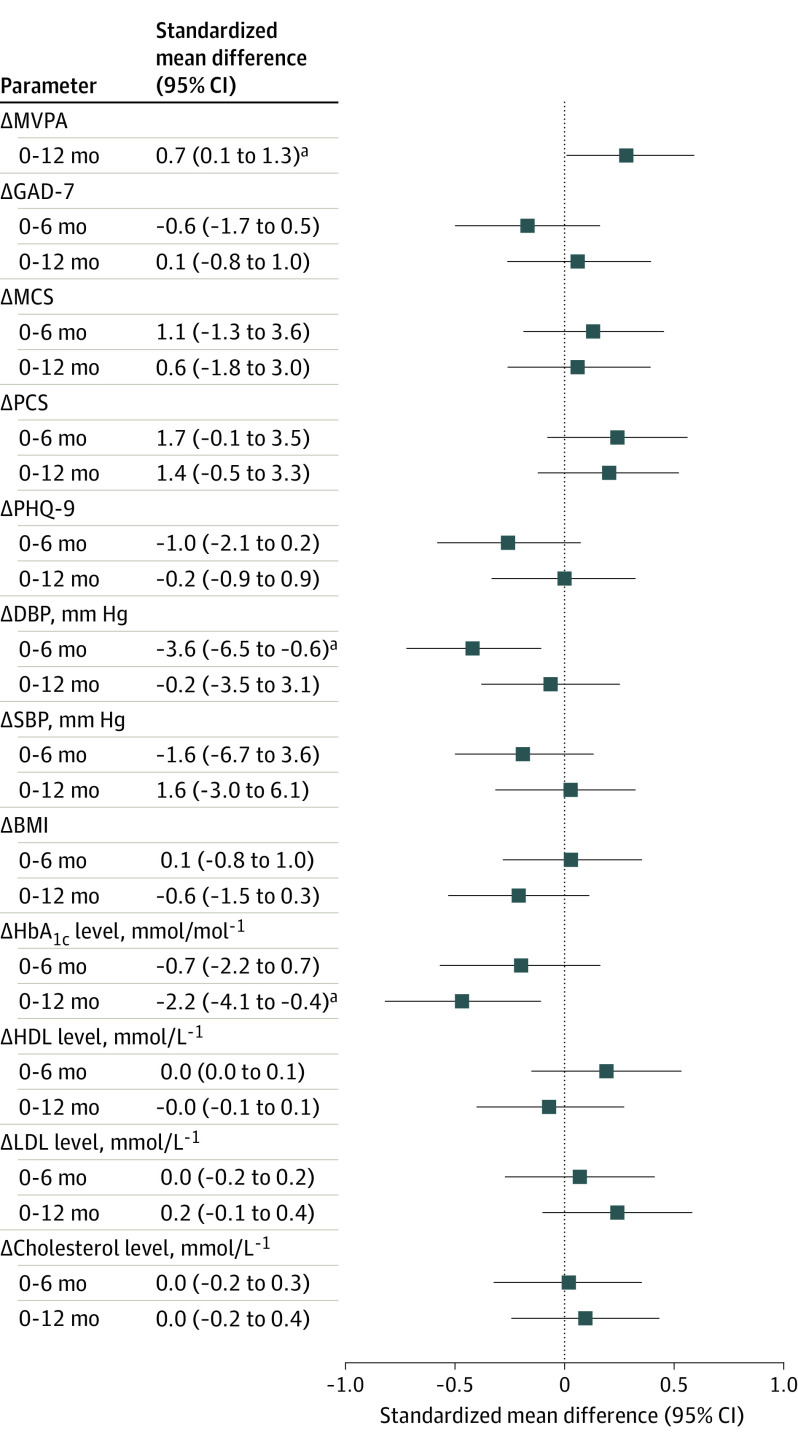
Standardized Mean Differences at 6- and 12-Month Follow-up in Secondary Outcome Parameters Compared with the control group, physical activity in days per week with more than 30 minutes of self-reported moderate to vigorous activity (MVPA) was significantly higher in the mobile cardiac rehabilitation (MCR) group; hemoglobin A_1c_ (HbA_1c_) level after 12 months increased more in the control group; and diastolic blood pressure (DBP) at 6 months was lower in the MCR group. BMI indicates body mass index; GAD-7, General Anxiety Disorder Questionnaire; HDL, high-density lipoprotein cholesterol; LDL, low-density lipoprotein cholesterol; MCS, mental component score of the 36-Item Short Form, version 2, survey (SF36v2); PCS, physical component score of the SF36v2; PHQ-9, Patient Health Questionnaire; and SBP, systolic blood pressure. ^a^*P* < .05.

Peak oxygen uptake increased after 6 months (1.6 [95% CI, 0.9 to 2.4] mL/kg^−1^/min^−1^; relative increase of 8.5%) and 12 months (1.2 [95% CI, 0.4 to 2.0] mL/kg^−1^/min^−1^; relative increase of 6.3%) for patients in the MCR group, whereas no changes were observed for the control group (6 months, +0.2 [95% CI, −0.4 to 0.8] mL/kg^−1^/min^−1^; 12 months, +0.1 [95% CI, −0.5 to 0.7] mL/kg^−1^/min^−1^) (eTable 2 in Supplement 2). Change in Vo_2_peak was higher in the MCR compared with the control group at 6 months (+1.2 [95% CI, 0.2 to 2.1] mL/kg^−1^/min^−1^) and 12 months (+0.9 [95% CI, 0.05 to 1.8] mL/kg^−1^/min^−1^) (Figure 2). Likewise, change in the amount of self-reported habitual physical activity was greater in the MCR compared with the control group (mean absolute difference, 0.7 [95% CI, 0.1-1.3]) (Figure 3). Diastolic blood pressure remained stable at 6 months in the MCR group (mean [SD] change from baseline, 78 [11] to 76 [12] mm Hg) and increased in the control group (mean [SD] change from baseline, 76 [12] to 78 [11]). In contrast, HbA_1c_ level increased in the control group at 12 months (mean [SD] change from baseline, 40.4 [5.7] to 42.0 [8.2] mmol/mol), whereas it remained stable in the MCR group (mean [SD] change from baseline, 42.6 [9.4] to 42.0 [8.1] mmol/mol) (eTable 2 in Supplement 2). Changes in other cardiovascular biomarkers did not differ between the MCR and control groups, and no differences were found in quality of life. The incidence of serious adverse events did not differ between the MCR and control groups (12 of 89 [13%] vs 10 of 90 [11%]; *P* = .66) (eTable 3 in Supplement 2). Most patients were hospitalized for acute (6 of 19 [3%]) or chronic (8 of 19 [42%]) coronary syndrome.

## Discussion

A 6-month home-based cardiac rehabilitation program for elderly patients who declined participation in conventional cardiac rehabilitation was beneficial in terms of change in physical fitness. These adaptations proved to be sustainable at 1-year follow-up. Moreover, MCR proved to be safe, because the incidence of adverse events was low and similar to rates observed in the control group. Patients in the MCR group demonstrated a greater increase in self-reported physical activity, whereas control patients had an increase in HbA_1c_ level. These observations indicate that MCR may be a valuable and safe tool for older patients with cardiac disease who decline hospital-based cardiac rehabilitation programs.

We found relative increases in physical fitness of 8.5% and 6.3% after 26 and 52 weeks, respectively. The magnitude of these improvements is beyond the coefficient of variation for assessment of Vo_2_peak (5.9%) and thus can be classified as genuine.^8,9^ Comparable populations showed a mean increase in exercise capacity of 16% (absolute, 2.52 [95% CI, 2.32-2.72] mL/kg^−1^/min^−1^)^10^ and 19% (absolute, from 19.4 [5] mL/kg^−1^/min^−1^ to 23.9 [6.0] mL/kg^−1^/min^−1^)^11^ 12 months after completion of a center-based cardiac rehabilitation program, whereas the present study found an increase of 6.3% (absolute, 1.2 [95% CI, 0.4-2.0] mL/kg^−1^/min^−1^). Potential explanations for the relatively smaller improvement in fitness in MCR may relate to differences in intensity of the training program and motivation of the patient. Center-based cardiac rehabilitation may lower anxiety for high-intensity training, allowing greater increases in Vo_2_peak.^12^ In addition, patients willing to participate in conventional cardiac rehabilitation might also be more motivated to reach exercise targets, leading to greater fitness improvements.^13^

Not only physical fitness but also secondary outcome parameters support the positive findings of MCR. Differences in secondary outcome parameters all favored MCR and may be associated with a higher increase in self-reported physical activity in MCR when compared with a control condition.^14^

Only 26% of eligible patients who declined center-based cardiac rehabilitation participated in the present study, which calls into question the clinical relevance and feasibility of MCR as a secondary prevention strategy. However, the potential of MCR may be higher because the EU-CaRE RCT was performed in countries with a relative high availability of cardiac rehabilitation programs and centers with a high participation rate.^15^ In regions with a lower availability of cardiac rehabilitation programs and a lower uptake of cardiac rehabilitation, more patients may benefit from MCR. Mobile cardiac rehabilitation can be a safe option for elderly patients who decline participating in conventional cardiac rehabilitation, because no differences were seen in adverse effects across both groups, and incidence of hospitalization in this vulnerable aged population was low.^16^

### Strengths and Limitations

The primary strength of the EU-CaRE RCT is that we were able to include a patient group not willing to participate in conventional center-based cardiac rehabilitation and thereby addressed an underrepresented group in traditional studies on cardiac rehabilitation. Moreover, by including different countries and different cultures, our results are more generalizable to the rest of the world. One limitation of the study is that we used MCR as an alternative for exercise-based cardiac rehabilitation and not for comprehensive cardiac rehabilitation, because we did not include all core components of cardiac rehabilitation in MCR. Of note, the EU-CaRE RCT was not powered to detect a difference in hard outcomes or more rare adverse events.

## Conclusions

In this RCT, a home-based MCR program of 6 months for elderly patients who decline participation in conventional cardiac rehabilitation was superior in changing physical fitness at 6- and 12-month follow-up when compared with usual care with no cardiac rehabilitation. Furthermore, MCR could be a safe alternative to improve fitness and increase physical activity in older patients. Future studies are warranted to explore the long-term clinical benefits of MCR in this patient group, including longevity, attenuated disease progression, and a reduced risk for adverse cardiovascular events.

## Notes

Supplement 1. **Trial Protocol**

Supplement 2. **eMethods.** Design and Participants **eFigure 1.** Flowchart of Study Protocol With All Contact Moments and Measurements **eFigure 2.** Main Reasons for Not Participating in Cardiac Rehabilitation **eTable 1.** Baseline Characteristics **eTable 2.** Primary and Secondary Outcome Parameters at Baseline and 6- and 12-Month Follow-up **eTable 3.** Adverse Events Within Study Period

Supplement 3. **Data Sharing Statement**
