# Supplementary material for: Effectiveness of Home-Based Mobile Guided Cardiac Rehabilitation as Alternative Strategy for Nonparticipation in Clinic-Based Cardiac Rehabilitation Among Elderly Patients in Europe: A Randomized Clinical Trial
Source: JAMA Cardiol. 2020 Oct 28;6(4):1–6. doi: 10.1001/jamacardio.2020.5218 (PMC7593879; doi:10.1001/jamacardio.2020.5218)
Supplement: Supplement 1. — Trial Protocol [file jamacardiol-e205218-s001.pdf]

Randomised Controlled Trial for an eHealth application in EUropean Cardiac Rehabilitation programmes in Elderly

EU-CaRE RCT

Assessment of the effects on sustainability and participation level of a mobile telemonitoring guided cardiac rehabilitation programme (*m*CR) and its cost effectiveness, in elderly patients in Europe

(July 2016)

Version 1.3

PROTOCOL TITLE '*Randomised Controlled Trial for an eHealth application in European Cardiac Rehabilitation programmes in Elderly; Assessment of the effects on sustainability and participation level of a mobile telemonitoring guided cardiac rehabilitation programme (mCR) and its cost effectiveness, in elderly patients in Europe' (EU-CaRE RCT*)

**Protocol ID**	**NL52862.075.15**
**Short title**	**Mobile telemonitoring guided cardiac rehabilitation programme (*m*CR) in elderly patients in Europe**
**Abbreviation of title**	**EU-CaRE RCT**
**Version**	**1.3**
**Date**	**08-07-2016**
**Principal investigator**	**Dr. E.P. De Kluiver (Department of Cardiology, Isala Zwolle, The Netherlands)**<br>e-mail: e.p.de.kluiver@isala.nl<br>phone: +31 (0)38 424 3073
**Site investigators**	**- Dr. E.P. De Kluiver (Isala Zwolle, The Netherlands)**<br>**- Dr. M.A. Brouwer (Radboudumc Nijmegen, The Netherlands)**<br>**- Prof. Dr. E. Prescott (Bispebjerg University Hospital Copenhagen (part of Region Hovedstaden) Denmark)**<br>**- Prof. J.R. Gonzalez-Juanatey (Servizo Galego de Saude, Spain)**<br>**- Dr. M. Wilhelm (University of Bern, Switzerland)**<br>**- Dr. M. Iliou (Assistance Publique – Hopitaux de Paris, France)**
**Sponsor**	**Dr. A.W.J. van 't Hof (Department of Cardiology, Isala Zwolle, The Netherlands)**<br>e-mail: a.w.j.vant.hof@isala.nl<br>phone: +31 38 4244361
**Subsidising parties**	**European Union; Government of Switzerland**
**Contract Research Organisation**	**Diagram B.V.**
**Planned start date:**<br>**Planned closing date:**	**01-07-2015**<br>**01-05-2019**

PROTOCOL SIGNATURE SHEET

Randomised Controlled Trial for an eHealth application European Cardiac Rehabilitation programmes in Elderly EU-CaRE RCT

I have read this protocol, appendices and amendment(s), if applicable, and agree to adhere to the requirements. I will provide copies of this protocol and all pertinent information to the study personnel under my supervision. I will discuss this material with them and ensure they are fully informed regarding set-up and conduct of the study.

I will conduct the study in accordance with the protocol, the Declaration of Helsinki, corresponding national laws, as well as local regulations, and I accept respective revisions to the protocol approved by authorised personnel of the Sponsor and by regulatory authorities.

Name	Name	Signature	Date
Sponsor:	Dr. A.W.J. van 't Hof (Department of Cardiology, Isala, Zwolle)		
Principal Investigator:	Dr. E.P. De Kluiver (Head Cardiology Department, Isala, Zwolle)		

PROTOCOL REVISION SUMMARY

Version	Release Date	Summary of Changes
1.0	13-03-2015	Initial Release
1.1	04-05-2015	Optimalisation protocol<br>Adjusted statistical paragraph<br>Adjusted inclusion - and exclusion criteria
1.2	11-06-2015	Lab<br>Questionnaire SF36
1.3	08-07-2016	Inclusion criteria<br>Mediterranean Diet Score<br>Statistical paragraph<br>Additional centre

TABLE OF CONTENTS

1. SUMMARY 8
2. INTRODUCTION AND RATIONALE 11
3. OBJECTIVES 12
4. STUDY DESIGN 14
5. STUDY POPULATION 15
5.1 Population (base) 15
5.2 Inclusion criteria 15
5.3 Exclusion criteria 15
5.4 Sample size calculation 16
6. TREATMENT OF SUBJECTS 17
6.1 Investigational product/treatment 17
6.2 Data collection by smartphone 18
7. METHODS 19
7.1 Study endpoints/outcomes and baseline characteristics 19
7.2 Study endpoints/outcomes 20
7.2.1 Patient related study endpoints/outcome 20
7.2.2 Training related outcomes 21
7.3 Patient characteristics 21
7.4 Centre characteristic 21
7.5 Randomisation, blinding and treatment allocation 21
7.6 Study procedures 22
7.7 Withdrawal of individual subjects 24
7.8 Replacement of individual subjects after withdrawal 24
7.9 Follow-up of subjects withdrawn from treatment 24
7.10 Follow-up of subjects withdrawn from study 24
7.11 Premature termination of the study 24
8. SAFETY 25
8.1 Section 10 WMO event 25
8.2 AEs, SAEs and SUSARs 25
8.2.1 Adverse events (AEs) 25
8.2.2 Serious adverse events (SAEs) 25
8.2.3 Adverse event reporting 26
8.2.3.1 Adverse event monitoring 26
8.2.3.2 Reporting to CRO and/or IRB/MEC/HREC 26
8.2.4 Suspected Unexpected Serious Adverse Reactions (SUSARs) 26
8.3 CLINICAL EVENT COMMITTEE 26
9. STATISTICAL ANALYSIS 27
9.1 Statistics 27
9.2 Primary analyses 27
9.3 Secondary analyses 28
9.4 Multivariable analysis 29

9.5 Population 29
9.6 Missing values 29
10. ETHICAL CONSIDERATIONS 30
10.1 Regulation statement 30
10.2 Recruitment and consent 30
10.3 Benefits and risks assessment, group relatedness 31
10.4 Compensation for injury 31
11. ADMINISTRATIVE ASPECTS, MONITORING AND PUBLICATION 32
11.1 Handling and storage of data and documents 32
11.2 Monitoring and Quality Assurance 33
11.3 Amendments 34
11.4 Annual progress report 34
11.5 End of study report 34
11.6 Public disclosure and publication policy 35
12. REFERENCES 36
13. APPENDIX I: Schedule of Events 38
14. APPENDIX II: Site investigator names and contact data per centre 39
15. APPENDIX III: Declaration of Helsinki 40

LIST OF ABBREVIATIONS AND RELEVANT DEFINITIONS

ACS	Acute coronary syndrome
AE	Adverse Event
CA	Competent Authority
CABG	Coronary artery bypass grafting
CAD	Coronary Artery Disease
CR	Cardiac rehabilitation
CRO	Clinical research organisation
CV	Curriculum Vitae
CVD	Cardiovascular Disease
EU	European Union
IC	Informed Consent
ICD	Implanted cardiac device
*m*CR	*mobile* telemonitoring guided cardiac rehabilitation
METC	Medical research ethics committee (MREC)
MI	Myocardial infarction
PCI	Percutaneous coronary intervention
RCT	Randomised controlled trial
(S)AE	(Serious) Adverse Event
Sponsor	The sponsor is the party that commissions the organisation or performance of the research, for example a pharmaceutical company, academic hospital, scientific organisation or investigator. A party that provides funding for a study but does not commission it is not regarded as the sponsor, but referred to as a subsidising party.
SUSAR	Suspected Unexpected Serious Adverse Reaction
TAVI	Transcatheter Aortic Valve Implantation
VO_{2peak}	Peak oxygen uptake or VO2 peak
Wbp	Personal Data Protection Act (In Dutch: Wet Bescherming Persoonsgegevens)
WMO	Medical Research Involving Human Subjects Act (In Dutch: Wet Medisch-Wetenschappelijk Onderzoek met mensen)

1. SUMMARY

Name of trial: Randomised Controlled Trial for an eHealth application in cardiac rehabilitation in Europe (EU-CaRE RCT)

Rationale:

Cardiovascular diseases (CVDs), such as coronary heart disease and stroke, are one of the four main non-communicable diseases in the world causing over 4 million deaths in Europe each year. Not only mortality rates are high, morbidity of CVD patients is becoming an increasingly important problem. Through enormous improvements in high-technology diagnostic and therapeutic procedures the survival rates from CVD in (Western) Europe have increased substantially. Yet, the recurrence rate of CVD events and consumption of care resulting from CVD, or associated co-morbidities are high and patient numbers are expected to rise the next decades due to an ageing population.

Literature shows that comprehensive cardiac rehabilitation (CR) is highly effective. However, knowledge on the effectiveness of individual CR components and their appropriateness for specific patient groups is limited. This is particularly true for the elderly with CVD, which represent a special population often characterised by physical impairment, comorbidities and reduced mobility. The current approach for CR is often less appropriate for the elderly, as a result of which effectiveness, compliance, participation levels and cost-utility of CR programmes in this group is hampered.

Home-based CR seems to be equally effective as centre-based CR and has the potential to increase the participation rate. In combination with novel e-Health applications (where guidance from distance is enabled), home-based care could overcome barriers to access to CR and therefore be a useful tool for increasing participation. Furthermore it seems that telehealth interventions are effective in improving self-management skills and provide effective risk factor reduction and secondary prevention. However, this is only shown on the short term. At this moment, long term effectiveness of telehealth interventions is still not known.

Therefore the aim of this study is to investigate whether a *mobile* telemonitoring guided CR (*m*CR) as alternative for a regular CR programme is an effective means to increase participation and adherence of elderly in a CR programme, and results in better sustained effects than in patients who do not follow the *m*CR programme. In addition the cost effectiveness of the *m*CR programme will be analysed.

Objectives: 1.To assess whether mobile telemonitoring guided CR (*m*CR) programmes result in better sustained effects on physical, mental and social outcomes in elderly patients not opting for regular CR, than patients who do not follow the *m*CR programme. 2. To assess whether *m*CR as alternative for a regular CR programme is an effective means to increase participation and adherence of elderly in a CR programme. 3. To analyse the induced costs of the *m*CR programme and determine its cost effectiveness.

Study design: Randomised controlled trial

Study Centres and Geography: 6 centres in several European countries

Study population: Elderly cardiac patients who are indicated for CR but not opting for regular CR

Sample size calculation: 238 patients in total (119 per study arm) divided over 6 centres, indicating approximately 40 patients per centre.

Intervention: Patients aged 65 years and older, who are candidate for CR, but nut opting for regular CR are randomised in two study arms: the *m*CR programme for 6 months or no *m*CR programme. The *m*CR programme involves a home-based programme for 6 months in which patients are supplied with a smartphone/application with a data subscription from MobiHealth. Through this application patients are able to measure and register physical activity, heart frequency and intensity (BORG scale) and can monitor progress. Patients are instructed to perform a moderate exercise 5 days per week for at least half an hour. A care professional (typically a CR nurse) also has access to a portal to monitor progress of different patients, advice on rehabilitation approach and stimulate compliance telephone calls. During the first month patients receive weekly individual coaching and feedback on their results by telephone, in the second month this will be once per two weeks, whereas one monthly call will be held in the last four months (month 3 until 6) of the mobile telemonitoring period. In the second period without mobile telemonitoring (month 7 until 12) patients will receive no coaching or feedback by phone. Patients participating in the control group with no *m*CR programme receive no advice or coaching throughout the study period.

Study endpoints/outcomes:

Main study endpoint/outcome

- Difference in peak oxygen uptake (VO_{2peak}) between the end of CR programme (T1) and baseline (T0)

Secondary study endpoints/outcomes

- Difference in VO_{2peak} between 12 months (T2) and T0
- Difference in VO_{2peak} between T2 and T1
- Traditional risk factors for CVD
- Major Adverse Cardiovascular Events (MACE)
- General health

- Care utilisation
- Costs of care utilisation
- Adherence
- Compliance

Patient follow-up: Patients will be monitored at T0-baseline, T1 – after 6 months and T2 – after 12 months.

Inclusion criteria: Patients aged 65 years or older who are candidate for CR and non-voluntary to participate in the regular CR programme, have signed a written consent and meet one of the following criteria: 1) acute coronary syndrome (ACS), including myocardial infarction (MI) and/or revascularisation within 3 months prior to the start of CR programme, 2) underwent percutaneous coronary intervention (PCI) within 3 months prior to the start of CR programme , 3) received coronary artery bypass grafting (CABG) within 3 months prior to the start of CR programme, 4) surgically or percutaneously treated for valvular heart disease including transcatheter aortic valve implantation (TAVI) within 3 months prior to the start of CR programme or 5) stable angina with documented significant coronary artery disease (CAD) (defined by standard non-invasive or invasive methods).

Exclusion criteria: Patients with a contraindication to CR, mental impairment leading to inability to cooperate, severe impaired ability to exercise, signs of severe cardiac ischemia and/or a positive exercise testing on severe cardiac ischemia, insufficient knowledge of the native language and an implanted cardiac device (pacemaker, ICD), no access, availability or insufficient knowledge of a computer with internet.

2. INTRODUCTION AND RATIONALE

Cardiovascular diseases (CVD), such as coronary heart disease and stroke, are one of the four main non-communicable diseases in the world causing over 4 million deaths in Europe each year. (1) Not only mortality rates are high; morbidity of CVD patients is becoming an increasingly important problem. The burden of CVD is not only huge for patients and the healthcare sector, but also encompasses a large economic strain with annual costs of €169 billion in the European Union (EU). Through enormous improvements in high-technology diagnostic and therapeutic procedures the survival rates from CVD in (Western) Europe have increased substantially. Yet, the recurrence rate of CVD events and consumption of care resulting from CVD, or associated co-morbidities are high and patient numbers are expected to rise the next decades due to an ageing population.(2)

Literature shows that cardiac rehabilitation (CR) is highly effective.(3-5) However, knowledge on the effectiveness of individual CR components and their appropriateness for specific patient groups is limited. This is particularly true for the elderly with CVD (6), which represent a special population often characterised by physical impairment, comorbidities and reduced mobility. As a result, the effectiveness, compliance, participation levels and cost-utility of CR programmes in this group is hampered.(5)

Home-based CR seems to be equally effective as centre-based CR and has the potential to increase the participation rate.(7, 8) In combination with novel e-Health applications (where guidance from distance is enabled), home-based care could overcome barriers to access to CR and therefore be a useful tool for increasing participation.(9) Furthermore it seems that telehealth interventions are effective in improving self-management skills and provide effective risk factor reduction and secondary prevention.(10-12) However, this is only shown on the short term, long term effectiveness of telehealth interventions is still not known.

Therefore the aim of this study is to investigate whether a *mobile* telemonitoring guided CR (*m*CR) programme as alternative for a regular CR programme is an effective means to increase participation and adherence of elderly in a CR programme, and results in better sustained effects than in patients who do not follow the *m*CR programme. In addition the induced and avoided costs and revenues of the *m*CR programme will be analysed in order to determine the cost effectiveness of this novel programme in comparison to other CR programmes.

3. OBJECTIVES

Objectives:

- To assess whether *m*CR programmes result in better sustained effects on physical, mental and social outcomes in elderly patients not opting for regular CR, than patients who do not follow the *m*CR programme.

- To assess whether *m*CR as alternative for a regular CR programme is an effective means to increase participation and adherence of elderly in a CR programme.

- To analyse the induced costs of the *m*CR programme and determine its cost effectiveness.

Main outcomes include:

- Physical fitness: difference in VO_{2peak} (T1-T0), (T2-T0) and (T2-T1)
- Traditional risk factors for CVD
- MACE
- General health
- Care utilisation
- Costs of care utilisation
- Adherence
- Compliance

The primary hypothesis of the study is that joining the *m*CR programme results in a greater difference in VO_{2peak} in cardiac patients between T1-T0 and T2-T0 than not joining the *m*CR programme.

The second hypothesis of the study is that joining the *m*CR programme results in an equal or greater difference in VO_{2peak} in cardiac patients between T2 and T1 than not joining the *m*CR programme.

The third hypothesis of the study is that the joining the *m*CR programme results in improved traditional risk profile (i.e. lipid profile, HbA1C, lean body mass, blood pressure) in cardiac patients in comparison to not joining the *m*CR programme after 6 (T1) and 12 months (T2).

The fourth hypothesis of the study is that the overall occurrence of MACE (cardiovascular mortality, all-cause mortality, near sudden cardiac death, acute coronary syndrome, CV

intervention/surgery, CV hospital admission, CV emergency visit) in the intervention group is lower than in the control group after T1 and T2.

The fifth hypothesis of the study is that joining the *m*CR programme results in an improved general health (e.g. quality of life (SF-36v2: Physical and Mental Component Summary Score), level of depression (PHQ-9), level of anxiety (GAD-7) and dietary pattern (Mediterranean Diet Score)) in comparison to not joining the *m*CR programme after T1 and T2 in cardiac patients.

The sixth hypothesis of the study is that utilisation of care in the intervention group is lower than in the control group throughout the study period.

The seventh hypothesis of the study is that the costs of care utilisation in the intervention group are lower than in the control group throughout the study period.

The eighth hypothesis of the study is that cardiac patients randomised to the *m*CR programme comply with the use of the smartphone (use of the smartphone for at least half an hour at 5 five days per week) during the first 6 months of the study.

4. STUDY DESIGN

The design used for this study is an open prospective, investigator initiated multicentre randomised controlled trial. A total of 238 cardiac patients (divided over six participating centres) suitable for CR (after approval cardiologist) and who do not volunteer to participate in the observational study will be included in this interventional study and randomly assigned by an algorithm to one of the 2 study groups, i.e. the 6 months of *m*CR programme, or 6 months of no *m*CR programme. Both groups have an additional 6 months of follow-up after the first period without any intervention. Measurements in both groups will be performed at T0, T1, and T2 (figure 1).

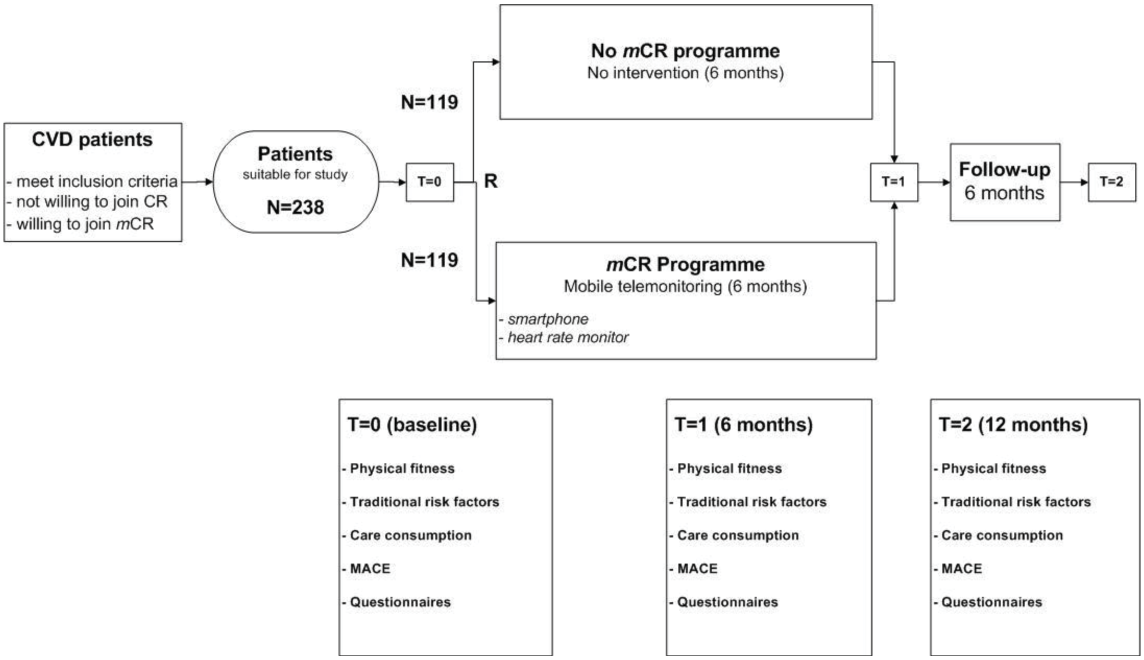

Figure 1. Study design divided over 6 centres.

5. STUDY POPULATION

5.1 Population (base)

A total of 238 patients (divided over six participating centres) meeting all inclusion criteria and none of the exclusion criteria will be included in this study. See paragraph 5.4 for sample size calculation.

5.2 Inclusion criteria

In order to be eligible to participate in this study, a subject must meet all of the following criteria:

- Patients of 65 years or older who are a candidate for CR and non-voluntary to participate in the regular CR programme
- Signed written informed consent
- One of the following criteria:
    - Patients with an acute coronary syndrome, including myocardial infarction (MI) and/or revascularisation within 3 months prior to the start of the CR programme
    - Patients that underwent a percutaneous coronary intervention (PCI) within 3 months prior to the start of the CR programme
    - Patients that received coronary artery bypass grafting (CABG) within 3 months prior to the start of the CR programme
    - Patients who were treated surgically or percutaneously for valvular heart disease (including TAVI) within 3 months prior to the start of the CR programme
    - Patients with a stable angina with documented significant CAD (defined by standard non-invasive or invasive methods)

5.3 Exclusion criteria

A potential subject who meets any of the following criteria will be excluded from participation in this study:

- Contraindication to CR
- Mental impairment leading to inability to cooperate
- Severe impaired ability to exercise
- Signs of severe cardiac ischemia and/or a positive exercise testing on severe cardiac ischemia
- Insufficient knowledge of the native language
- No access, availability or insufficient knowledge of a computer with internet
- Implanted cardiac device (pacemaker, ICD)

5.4 Sample size calculation

In order to assess the effects on sustained physical fitness after participation in the *m*CR programme it was determined that an inclusion of 119 patients per group would be sufficient to examine the aim of this study.

This is based on the expected difference in increase in VO_{2peak} level of 3.0 ml/kg/min at 6 months between the control and intervention group with an estimated standard deviation of 5 ml/kg/min in the intervention group and 6 ml/kg/min in the control group. Assuming 80% power and 5% two-sided significance level (α=0.05), the sample size (n) required to achieve a probability of 80% of detecting a difference in an increase VO_{2peak} level between two independent groups is n=55 per randomisation group. Since patients from different centres (countries) will be pooled in the analysis, we will apply an adjustment for dependency of the observations to calculate the sample size, based on an estimated intraclass correlation coefficient (ICC) for the study centres.

The ICC is the proportion of the total variance which is between the centers rather than within centres. An ICC of 0.05 is used in the sample size calculations. After adjustment for the ICC the sample size is N=83 per group. Based on 30% withdrawal we aim for a group size of n=119 per randomisation group (n=238 in total). Six centres will participate in the RCT so we will aim at including approximately 40 patients per centre. As this is a cooperative trial, centres may recruit more patients than this recruitment goal. When one centre starts to dominate enrolment however, recruitment might need to be capped. The total sample size (n=238) would be sufficient to detect a clinically and physiologically relevant change in peak oxygen consumption, despite a 30% dropout.

6. TREATMENT OF SUBJECTS

6.1 Investigational product/treatment

Patients participating in the *m*CR programme (or intervention group) will undergo 6 months of mobile telemonitoring guidance and in addition another 6 months without mobile telemonitoring. The *m*CR group receives a heart rate monitor and smartphone with a special application to use for their training in their home environment. This smartphone can only be used by the patient for research purposes and will be given back to the researcher once the patient finishes the first 6 months in the study with mobile telemonitoring guidance. Instructions are given to the patient by the researcher, nurse or physician before they start training with a heart rate monitor and smartphone at home. Patients are instructed to exercise while wearing the heart rate monitor at 5 days per week for at least half an hour at an individual selected level of intensity and self-chosen type of activity. The outcomes of the maximal exercise tests on the first visit will enable to personalise the heart rate zones per patient in order to see (after every training/activity) in what zone the patient is training. At the moment patients start an exercise, they need to wear the heart rate monitor, and select on their smartphone the type of activity they are going to perform. While finishing the exercise patients have to register at the smartphone they are done and fill in the rate of perceived exertion (BORG-score). Accordingly, the duration, intensity (based on the heart rate zones) and BORG-score of each training/activity is collected by the smartphone and transferred to a secured website where both patient and researchers/nurses involved in the study can view the results. During the first month patients receive weekly individual coaching and feedback on their training results by telephone, in the second month this will be once per two weeks, whereas one monthly call will be held in the last four months (month 3 until 6) of the mobile telemonitoring period. Mobile telemonitoring guidance will help to improve the self-management skills and stimulate a patient to exercise independently. Besides the coaching by telephone and the advice to train 5 days a week for a minimum of 30 minutes at the start of the first 6 months, there will be no other advices given by the researcher, nurse or physician via the smartphone or secured website. At the end of the first 6 months, the patients are asked to return the heart rate monitor and smartphone to the researcher. In the second period without mobile telemonitoring (month 7 until 12) patients will receive no coaching or feedback by phone. Cardiac patients that are randomised to the control group will undergo 6 months with no *m*CR programme with another 6 months of follow-up in addition. During the study period both groups will receive standard outpatient care and control by a cardiologist. See paragraph 6.2 for more information about the way data are collected by the smartphone in the intervention group.

6.2 Data collection by smartphone

Patients in the intervention group receive a heart rate belt and smartphone with a self-tracking application of MobiHealth which is able to measure physical activities. With this application patients can register the duration and type of activity they are about to perform from a predefined list (i.e. cycling, walking, et cetera) and subjective perceived exertion (BORG score) afterwards. The wireless connected heart rate belt measures the heart rate of the patient during the activity which will subsequently be transferred to the smartphone application by Bluetooth. Both patient and researchers/nurses/physicians involved in the study have access to a MobiHealth Rehabilitation™ portal where data is presented with regards to their personal guidelines set for activities. A personal guideline regarding the intensity and frequency for the self-chosen activity for the patient is entered once at the start of the study by the researcher/nurse/physician. MobiHealth has a CE marking for its software.

7. METHODS

7.1 Study endpoints/outcomes and baseline characteristics

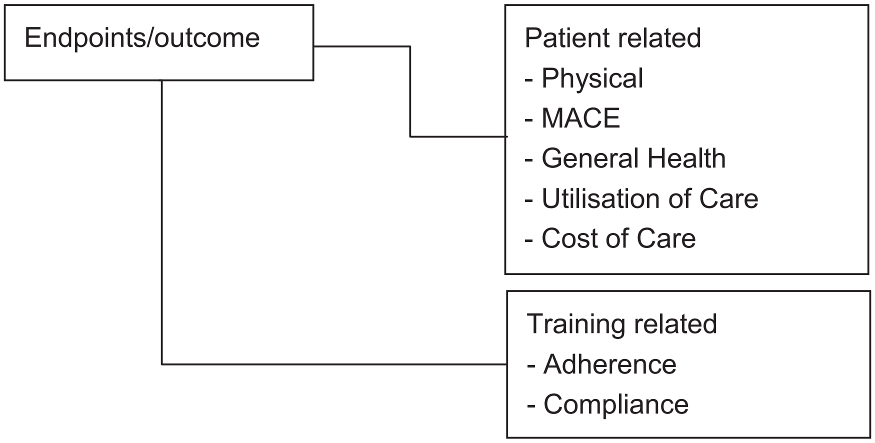

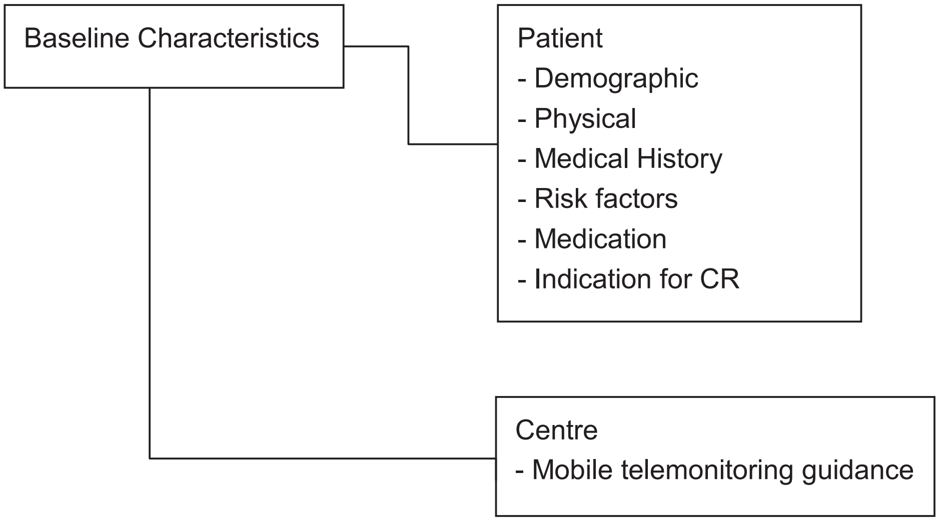

7.2 Study endpoints/outcomes

7.2.1 Patient related study endpoints/outcome

Main study endpoint

- Difference in VO_{2peak} from an incremental exercise test (T1-T0)

Secondary study endpoints/outcome

- Difference in VO_{2peak} from an incremental exercise test (T2-T0, T2-T1)

- Traditional risk factors:
  - Changes in lipid profile (T1-T0, T2-T0, T2-T1)
  - Changes in HbA1C (T1-T0, T2-T0, T2-T1)
  - Changes in renal function (T1-T0, T2-T0, T2-T1)
  - Changes in lean body mass (T1-T0, T2-T0, T2-T1)
  - Changes in blood pressure (T1-T0, T2-T0, T2-T1)
  - Changes in smoking habit (T2-T0)

- MACE: the occurrence of events (cardiovascular (CV) mortality, all-cause mortality, near sudden cardiac death, ACS, CV intervention/surgery, CV hospital admission, CV emergency visits) as composite endpoint (T2-T0) are registered and collected by monthly telephone calls.

- General health:
  - Difference in depression score assessed by: PHQ-9 questionnaire (T1-T0, T2-T0, T2-T1)
  - Difference in anxiety score assessed by GAD-7 questionnaire (T1-T0, T2-T0, T2-T1)
  - Quality of Life: SF-36v2, difference in Physical Component Summary Score and Mental Component Summary Score (T1-T0, T2-T0, T2-T1)
  - Difference in dietary pattern (reflected by total score and score on individual components) assessed by Mediterranean Diet Score (T1-T0, T2-T0, T2-T1)

- Care utilisation as composite endpoint of: (number of) admissions, emergency visits and cardiac interventions (PCI, CABG) (T2-T0).

- Costs of care utilisation based on activities (clinical admission days, emergency and outpatient clinic visits, GP visits for cardiac (related) complaints or issues, radiology/cardiophysiology/nuclear and laboratory tests,

and cardiac interventions) registered at T0, T1 and T2 and collected by monthly telephone calls with the participants between T1 and T2.

7.2.2 Training related outcomes

- Adherence (number of drop-out or completed CR) throughout study period.

- Compliance: compliance to usage of the smartphone (percentage of fulfilling the planned exercise sessions with *m*CR for at least half an hour at 5 five days per week) in the intervention group is determined for the period between baseline and 6 months.

7.3 Patient characteristics

- Demographic data: gender, age, educational attainment, ethnicity, working
- Physical parameters weight, length, BMI, lean body mass
- Medical history:
  - Cardiovascular
  - Co-morbidity
  - Current complaints (angina, dyspnea)
- Risk factors
- Medication (T0, T1, T2)
- Indication for CR
- Exercise test parameters (T0, T1, T2)
- Echocardiographic parameters (LV function)

7.4 Centre characteristic

- Mobile telemonitoring guidance: the coaching by telephone is performed by a physician, nurse or researcher.

7.5 Randomisation, blinding and treatment allocation

At the start of the CR programme, once patients have indicated not to be interested in the regular CR programme, patients will be informed about the study and requested to participate. After receiving written consent from the patient they will be randomised in 1:1 ratio to the intervention group with mobile telemonitoring guidance or the control group with no mobile telemonitoring guidance or other CR programme by means of computerised allocation system applying an algorithm that prevents the care providers or the investigators from predicting the outcome of the randomisation

process. This means that the person, who determines if the patient is eligible, at the time of this decision, is unaware of which group the patient will be allocated to. The patient, care provider and outcome assessor will not be blinded to the intervention itself because of safety concerns.

7.6 Study procedures

After screening (medical history, physical examination including blood pressure, laboratory check, ECG, questionnaire [general health, disease history, physical activity, lifestyle]), and approval of the cardiologist, patients will be included in the study. Patients will undergo several tests at one testing day. Testing will be performed at T0, T1 and T2 (figure 2).

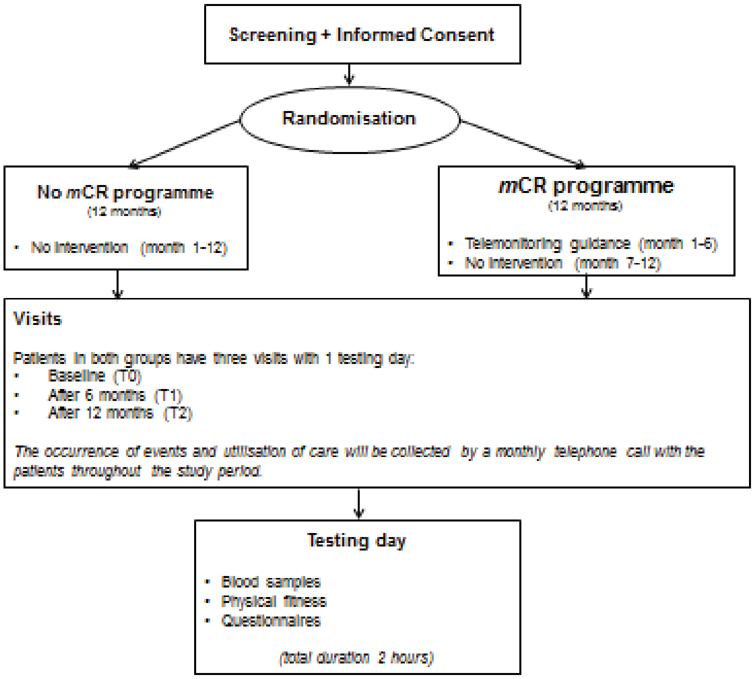

Figure 2. Study procedures

<u>Testing day: Blood samples, Physical Fitness and Questionnaires (2 hours)</u> (See Appendix I-Schedule of events)

Blood samples

Blood samples will be obtained for the analyses of a number of CVD risk parameters (e.g. HDL, LDL, HbA1C) and renal function.

Physical fitness

Before the assessment of physical fitness, skinfold thickness will be determined (biceps, triceps, sub-scapular, supra-iliac) to calculate the lean body mass. (13)

Physical fitness level will be measured as the VO_{2peak} during an incremental maximal exercise test, using a continuous gas analyser. ECG and heart rate will be registered continuously and blood pressure will be measured at fixed intervals of 3 minutes. The test will be performed under supervision. The modality for the exercise test will be cycling. All tests will be performed by the same protocol. For patients who are not able to participate in a VO_{2peak} test, this test will be replaced by a 6 Minutes Walking Test. The testing modality chosen per centre at T0, will remain the same at T1 and T2. To ensure a safe environment in which the tests can be performed, the test will be performed to applicable guidelines and regulations within each participating centre.

Questionnaires

After the incremental maximal ergometer exercise test, subjects will be asked to fill out four validated questionnaires to assess general health. This includes quality of life (SF-36v2), level of anxiety (GAD-7), level of depression (PHQ-9) and dietary pattern (Mediterranean Diet Score) of the patient. (14-20) These postal questionnaires will be completed at T0, T1 and T2. All questionnaires are easily completed within 30 minutes.

Information Collection MACE, Care Consumption

The occurrence of events (MACE (21) [cardiovascular mortality, all-cause mortality, near sudden cardiac death, acute coronary syndrome, CV Intervention/surgery, CV hospital admission, CV emergency visits]) and utilisation of care ((days) admission, outpatient clinic visits, GP visits, interventions, cardiophysiology, radiology, nuclear and lab testing) will be collected by a monthly telephone call with the participants using a standardised schedule throughout the study period. The telephone calls will be performed by a nurse. No advices or support will be given to the participants regarding their daily exercise or lifestyle changes.

7.7 Withdrawal of individual subjects

Patients can leave the study at any time for any reason if they wish to do so without any consequences. The site investigator can decide to withdraw a patient from the study for urgent medical reasons.

7.8 Replacement of individual subjects after withdrawal

We will not replace patients after withdrawal of the study.

7.9 Follow-up of subjects withdrawn from treatment

Patients in the study who experience a reintervention and get a new indication for CR will still be tested at 6 months and 12 months according to the original schedule. However, patients participating in the intervention group perceive no mobile telemonitoring guidance anymore. All patients will also be followed on major outcomes; relapse of myocardial infarction, death, PCI and CABG and utilisation of care.

7.10 Follow-up of subjects withdrawn from study

Patients who leave the study prematurely without any reason or because of an experienced reintervention with a contraindication to CR as a consequence will be followed on major outcomes; relapse of heart infarct, death, PCI and CABG and utilisation of care.

7.11 Premature termination of the study

Only in case of logistical reasons the EU-CaRE consortium (steering committee) can decide to end the study prematurely.

8. SAFETY

8.1 Section 10 WMO event

In accordance to section 10, subsection 1, of the WMO, the site investigator will inform the subjects and the reviewing accredited METC if anything occurs, on the basis of which it appears that the disadvantages of participation may be significantly greater than was foreseen in the research proposal. The study will be suspended pending further review by the accredited METC, except insofar as suspension would jeopardise the subjects' health. The site investigator will take care that all subjects are kept informed.

8.2 AEs, SAEs and SUSARs

8.2.1 Adverse events (AEs)

Adverse events are defined as any undesirable experience occurring to a subject during the study, whether or not considered related to the experimental intervention. All adverse events reported spontaneously by the subject or observed by the site investigator or his staff will be recorded.

8.2.2 Serious adverse events (SAEs)

A SAE is any untoward medical occurrence or effect that at any dose:

- results in death;
- is life threatening (at the time of the event);
- requires hospitalisation or prolongation of existing inpatients' hospitalisation;
- results in persistent or significant disability or incapacity;
- is a congenital anomaly or birth defect;
- Any other important medical event that may not result in death, be life threatening, or require hospitalisation, may be considered a serious adverse experience when, based upon appropriate medical judgement, the event may jeopardise the subject or may require an intervention to prevent one of the outcomes listed above.

8.2.3 Adverse event reporting

8.2.3.1 Adverse event monitoring

The site investigator will monitor the occurrence of AEs for each subject during the course of the study. All AEs reported by the subject, observed by the site investigator, or documented in medical records will be recorded on the AE sheet in the e-CRF, whether believed by the site investigator to be related or unrelated to the study. Starting with the study enrolment, any new event/experience that was not present at T0, or worsening of an event presents at T0, is considered an AE. AEs will not be followed up on once patients finish the study after 12 months. Unchanged, chronic conditions are not AEs and should not be recorded on the AE sheet in the e-CRF. SAEs will be collected and monitored throughout the entire course of the study.

8.2.3.2 Reporting to CRO and/or IRB/MEC/HREC

Each AE or complication meeting the definition for SAE will be reported upon discovery to the Clinical Research Organization (CRO), preferably within 24 hours of the site investigator's knowledge of the event. The site investigator will further report the event annually to its own institution. The subject's course must be monitored until the event has subsided or, in a case of permanent impairment, until the event stabilises and the overall clinical outcome has been ascertained.

8.2.4 Suspected Unexpected Serious Adverse Reactions (SUSARs)

SUSARs are not expected to occur to a subject during this study.

8.3 CLINICAL EVENT COMMITTEE

The Clinical Event Committee (CEC) will review and adjudicate all clinical endpoint events. The role, membership and meeting schedule of the CEC together with a definition and procedure of the endpoints to be reviewed will be stated in a separate document.

9. STATISTICAL ANALYSIS

9.1 Statistics

The study parameters are continuous and categorical variables. Categorical variables will be summarised by frequency and percentages. Continuous variables will be summarised by mean and standard deviation (sd) as well as median and interquartile range (IQR). The study parameters will be presented by centre, by intervention group and by time point (baseline, 6 months and 12 months follow-up).

We will describe the differences in outcomes over time between intervention groups in tables and graphs. All endpoints will be analysed for effects related to the intervention (T1) and sustainable effect (T2). Dropout and levels of compliance will be computed and presented as a total, by centre and by intervention group. (Costs of) health care utilisation will be described by centre, by intervention group and by visit. Scores on the self-report validated questionnaire will be coded and summarised according to the corresponding manuals.

The primary treatment comparison is *m*CR group versus control group. Student's t-tests will be used to examine whether the continuous variables are different between the intervention groups at 6 months and at 12 months. When necessary the continuous variables will be transformed to obtain a normal distribution, or a non-parametric test will be used. X2 tests or Fisher's exact tests as appropriate will be used to examine whether the categorical variables are different between the intervention groups at 6 months and at 12 months. A two-sided p-value of less than 0.05 will be considered to be statistically significant in all analyses.

Statistical analyses will be performed in SPSS and SAS. The statistical analyses will be described in detail in the Statistical Analysis Plan.

9.2 Primary analyses

The primary endpoint is the change in VO_{2peak} level between baseline and at 6 months. It will be tested whether this change is different in the *m*CR and the control group by means of linear mixed models with the intervention as a fixed effect. The effect of centre on the outcome is modelled with a random effect. In the analyses we will adjust for the baseline value of VO_{2peak}. When necessary the outcome variable will be transformed to obtain a normal distribution. The null hypothesis is that there is no difference in mean change in VO_{2peak} level between the randomised groups. The alternative hypothesis is that the mean

change in VO_{2peak} level differs between the randomised groups. A two-sided p-value of less than 0.05 will be considered to be statistically significant.

9.3 Secondary analyses

Sustainable effectiveness

Sustainable effectiveness is defined as a significant improvement in VO_{2peak} level from baseline (T0) to 12 months after start of CR programme (T2). Differences between intervention groups in sustainable effect will be analysed in similar linear mixed models comparing the difference between change in VO_{2peak} from T0 to T2 adjusting for baseline values of VO_{2peak}.

Differences between intervention groups in any of the outcome measures over time will be examined using mixed models repeated measures analysis (MMRM) with intervention group and time points as fixed effects and centre as random effect and baseline values as covariate. When necessary the outcome variables will be transformed to obtain a normal distribution. The null hypothesis is that the outcome under study is the same at all time points (T0, T1 and T2). The alternative hypothesis is that the outcome under study at at least one of the time points differs from the others. We will test for the interaction between intervention and time in order to investigate whether the development of study parameters over time differs between interventions. A two-sided p-value of less than 0.05 for a coefficient will be considered to be statistically significant. If the results of the MMRM analysis are statistically significant, we will run post hoc tests to highlight where these differences occur, adjusting the p-value for multiple comparisons using the Tukey procedure.

Differences between intervention groups in utilisation of medical care will be tested by linear mixed models with centre as random effect for continuous outcome or mixed models for logistic regression for dichotomous outcomes. When necessary continuous outcome variables will be transformed to obtain a normal distribution.

The cumulative incidences of the composite endpoint MACE and the elements from which it is constituted will be presented by intervention group. We will compute the probability of survival free from MACE past given time points (6 months and 1 year) and the survival function will be plotted by intervention group in Kaplan-Meier survival curves. The Kaplan Meier curves will indicate the occurrence of MACE during 1 year of follow-up. Patients who do not experience an event or are lost to follow-up without an event are censored.

We will test for differences in hazard rates between the *m*CR and control group using Cox regression analysis allowing the baseline hazard functions to differ between centres.

A two-sided p-value of less than 0.05 will be considered to be statistically significant in all univariate analyses.

9.4 Multivariable analysis

In case the groups differ on baseline values or other possibly confounding variables, the difference in change in VO_{2peak} level between baseline and 6 months between the intervention groups will be tested by means of linear mixed models analysis with centre as a random effect in which we will control for relevant confounders by adding covariates to the model. We will conclude a statistical significant difference in change in VO_{2peak} level between the *m*CR and control group when the two-sided p-value of the test statistic after adjustment for confounders is less than 0.05.

9.5 Population

The intention to treat population is used for analysis of the primary and secondary endpoints. All patients will be analysed in the group they are randomised to and whether or not they completed the *m*CR programme according to protocol.

9.6 Missing values

We will present numbers and percentages of missing values. Missing values will be analysed in order to see if these missing values are systematically different between both groups (*m*CR and control group) or whether they are related to baseline values or values on earlier time points. In case that the occurrence of missing values is systematically different between groups, or is related to other relevant variables, multiple imputation methods will be used to calculate parameter estimates as a sensitivity analysis.

10. ETHICAL CONSIDERATIONS

10.1 Regulation statement

All patients participating in the clinical study are extensively informed with the help of the patient information about the planned examinations as well as their benefits and risks. The patient consents to participating in the study and the forwarding of his or her data by signing the respective form.

A central vote is applied for at the ethics committee responsible for the principal investigator. The study can only begin after a positive vote.

All examinations are performed by the site investigator or a co-investigator authorised by him or her in accordance with the Declaration of Helsinki (see APPENDIX II), the corresponding national laws, and according to the current state of science and technology.

Any patient-related information that reaches the sponsor during this study is treated confidentially. Only specially authorised and trained personnel are given access to the study data. The stipulations recommended in the Declaration of Helsinki and required in national laws regarding data protection comply with by all involved parties.

10.2 Recruitment and consent

Patients will be recruited from the department of Cardiology of each participating centre. A physician or nurse specialised in CR will screen the patients and decide whether they can be a potential participant in this study. All patients who participate in this study have an adequate insurance coverage after weighing all risks (patient insurance). All patients participating in the clinical study are screened and extensively informed by a physician or nurse specialised in CR with the help of the patient information about the planned examinations as well as their benefits and risks. The patient consents to participating in the study and the forwarding of his or her data by signing the respective form.

Any patient-related information that reaches the sponsor during this study is treated confidentially. Only specially authorised and trained personnel are given access to the study.

Patients, who are capacitated, meet the inclusion and exclusion criteria and sign the informed consent, will be considered enrolled in the study. In case of incapacity of the patient, he or she is not able to participate in the study. Informed consent forms have to be in compliance with the latest Declaration of Helsinki. The patient shall be given a copy of the signed informed consent form, and the original shall be kept in the centre’s regulatory file.

10.3 Benefits and risks assessment, group relatedness

At all participating centres, there is a long-standing tradition in performing non-invasive testing as used in this present study. All procedures are performed routinely at the departments and have been accepted by the ethics committee in numerous previous applications in healthy as well as various patients groups.

Non-invasive cardiac testing procedures in this study are not related to any potential risk for the participant. Maximal ergometer tests will be performed at the hospital under supervision of highly qualified personnel.

A possible complication of venipuncture is a hematoma, which is induced in ~5% of all cases. To prevent complications, an experienced professional will perform the blood withdrawal and sufficient pressure will be provided after withdrawal of the needle. As patients are carefully instructed beforehand and individually coached by telephone on their physical progress during the first 6 months of the study we expect no potential risk for them to exercise in their home environment.

Taken together, our study involves minimally and non-invasive measures, whilst the *m*CR programme is believed to have a strong and potent health benefit for cardiac patients, and may eventually results in sustained effect on physical and mental health outcomes.

10.4 Compensation for injury

DIAGRAM maintains appropriate clinical trial liability insurance coverage as required under applicable laws and regulations and will comply with applicable local law and custom concerning specific insurance coverage. All patients participating in the study are insured against damages according to the regulation.

11. ADMINISTRATIVE ASPECTS, MONITORING AND PUBLICATION

11.1 Handling and storage of data and documents

Electronic Case Report Forms (eCRFs) have been developed to capture the study information outlined in this protocol. Data from these eCRFs will be used in the analysis of study results. Modification of the eCRFs will only be made if deemed necessary by the principal investigator.

All centre personnel will be trained on the protocol and internet-based database eCRF prior to the initiation of the study. Trained and qualified Clinical Research Associates (CRAs) of Diagram and/or his designee will monitor the trial throughout its duration by means of personal visits to the physician's facilities, telephone contacts to the physician or designee, and/or remotely through the internet-based database. A comprehensive, integrated data management plan, including on-line queries and remote data cleaning will be implemented to insure the integrity of the data. All changes to the database will be tracked by an audit trail.

The site investigator, or a designated individual, is responsible for recording all data from the study on the eCRFs based on source-documented hospital chart reviews at his/her centre. The site investigator is required to electronically sign the eCRF on the appropriate pages to verify that he/she has reviewed and attests to the correctness of the recorded data.

All eCRFs should be thoroughly completed within 30 days after the study staff study specific information. For adverse event reporting, refer to section on Adverse Event Reporting Requirements and timelines (see paragraph 7.2).

Data entered in to the eCRFs will be taken from source documentation, such as hospital procedure reports, admission and discharge summaries and other hospital or physician office/clinic documents. If no standard hospital or office document exists to capture some of the information that may be unique to this study, a worksheet may be developed to record this information, which needs to be signed by the site investigator at the given centre, and which will serve as the source document for those data parameters. These source documents will serve as the basis for monitoring the eCRFs. Electronic patient records will be considered as source documents on the condition that the hospital's database is a validated system. If this is not the case, electronic records will have to be printed and added to the paper patient file. A print-out of the eCRF cannot be used as source documentation.

The site investigator will maintain all records pertaining to this study for fifteen years following study completion, or as otherwise instructed by the CRO, or per local requirements whichever is longer.

11.2 Monitoring and Quality Assurance

Source data verification will be done completely for at least the first five included patients per centre and 10% of the other included patients. For the other included patients at least informed consent and reported endpoints will be checked by the CRA. The site investigator will permit access to such records. Source documentation must be available to substantiate proper informed consent procedures, adherence to protocol procedures, adequate reporting and follow-up of adverse events, accuracy of data collected on case report forms, and device information. A monitoring visit sign-in log will be maintained at the centre. The site investigator and/or research coordinator will be available for monitoring visits. It is expected that the site investigator will provide the study monitor with a suitable working environment for review of study-related documents.

Investigators Responsibilities

The site investigators at the clinical centre will have the following responsibilities:

- Obtaining approval from the Medical Ethics Committee
- Obtaining written informed consent from patients
- Adequate enrolment of patients
- Performing medical procedures
- Adherence to the clinical protocol
- Following patients according to the protocol

Protocol Compliance

The site investigator is responsible for monitoring compliance with the clinical investigation protocol. The CRA will perform visits to inspect and audit study files and regulatory documentation, including site investigator and co-investigator curricula vitae, Ethics Committee approval documentation, patient informed consent forms and study correspondence logs. When problems are identified, assistance will be given to the appropriate individuals to ensure consistency of data collection procedures and transmission of data forms to the investigator. The site investigator will verify that the data recorded on the procedure forms are correct through review of reports and medical records. CRA and/or designee will monitor the study over its duration according to the pre-specified monitoring plan. The CRA will visit each centre at appropriate intervals to review investigational data for accuracy and completeness and ensure compliance with the protocol. The CRA may inspect all documents and required records that are maintained by the site investigator, including medical records (office, clinic or hospital) for the subjects in this study. The site investigator will permit access to such records. Source documentation must be available to substantiate proper informed consent procedures,

adherence to protocol procedures, adequate reporting and follow-up of adverse events, accuracy of data collected on case report forms, and device information. A monitoring visit sign-in log will be maintained at the centre. The site investigator and/or research coordinator will be available for monitoring visits. It is expected that the site investigator will provide the study monitor with a suitable working environment for review of study-related documents.

Name and Address of CRA
Diagram B.V.
Dokter Stolteweg 96
8025 AZ Zwolle
The Netherlands
+31 (0)38-4262999

11.3 Amendments

Amendments are changes made to the research after a favourable opinion by the accredited METC has been given.

11.4 Annual progress report

The site investigator will submit a summary of the progress of the trial to the accredited METC once a year. Information will be provided on the date of inclusion of the first subject, numbers of subjects included and numbers of subjects that have completed the trial, serious adverse events/ serious adverse reactions, other problems, and amendments.

11.5 End of study report

The site investigator will notify the accredited METC of the end of the study within a period of 8 weeks. The end of the study is defined as the last patient’s last visit.

In case the study is ended prematurely, the site investigator will notify the accredited METC within 15 days, including the reasons for the premature termination.

Within one year after the end of the study, the site investigator will submit a final study report with the results of the study, including any publications/abstracts of the study, to the accredited METC

11.6 Public disclosure and publication policy

The publication rights in regard to the main results of the study, i.e., regarding the primary and secondary objectives, belong to the principal investigator. Additional authors are determined by the volume and quality of data at the remaining investigational centres. All site investigators who hold a role of average importance with respect to the volume and quality of data are taken into account for the publication of the essential results of the study.

Additional publications of the analysis results of all patient data can be originated by the investigator as primary author whose ideas are behind a specific paper. However, he/she must at least hold a role of average importance with respect to the volume and quality of data. However, the decision about primary authorship is made by the principal investigator in consultation with the clinical project manager. The primary author then decides with the principal investigator and the involved investigators on the order of additional authors.

The principal investigator and project coordinator have to decide on the publication of data from just one study centre or a selection of investigational centres. The latter publications might have to wait until the publications based on all data have been written.

The primary author writes all publications. If desired, the primary author is supported by the clinical project manager. Before the release of a publication, it must be constructively reviewed and released by all authors. The sponsor will have the chance to review any abstract, manuscript before submission or presentation.

Every investigator can refuse to become a primary or co-author, exchange his or her authorship with another investigator from his or her investigational centre, or exchange his or her primary authorship with a co-authorship.

12. REFERENCES

1. Cardiology EHNaESo. European Cardiovascular Disease statistics 2012 edition. September 2012.
2. Murray CJ, Lopez AD. Alternative projections of mortality and disability by cause 1990-2020: Global Burden of Disease Study. Lancet. 1997;349(9064):1498-504. Epub 1997/05/24.
3. Clark AM, Hartling L, Vandermeer B, McAlister FA. Meta-analysis: secondary prevention programs for patients with coronary artery disease. Annals of internal medicine. 2005;143(9):659-72. Epub 2005/11/03.
4. Taylor RS, Brown A, Ebrahim S, Jolliffe J, Noorani H, Rees K, et al. Exercise-based rehabilitation for patients with coronary heart disease: systematic review and meta-analysis of randomized controlled trials. The American journal of medicine. 2004;116(10):682-92. Epub 2004/05/04.
5. European Association of Cardiovascular P, Rehabilitation Committee for Science G, Eacpr, Corra U, Piepoli MF, Carre F, et al. Secondary prevention through cardiac rehabilitation: physical activity counselling and exercise training: key components of the position paper from the Cardiac Rehabilitation Section of the European Association of Cardiovascular Prevention and Rehabilitation. European heart journal. 2010;31(16):1967-74. Epub 2010/07/21.
6. Ferrara N, Corbi G, Bosimini E, Cobelli F, Furgi G, Giannuzzi P, et al. Cardiac rehabilitation in the elderly: patient selection and outcomes. The American journal of geriatric cardiology. 2006;15(1):22-7. Epub 2006/01/18.
7. Oerkild B, Frederiksen M, Hansen JF, Simonsen L, Skovgaard LT, Prescott E. Home-based cardiac rehabilitation is as effective as centre-based cardiac rehabilitation among elderly with coronary heart disease: results from a randomised clinical trial. Age and ageing. 2011;40(1):78-85. Epub 2010/09/18.
8. Oerkild B, Frederiksen M, Hansen JF, Prescott E. Home-based cardiac rehabilitation is an attractive alternative to no cardiac rehabilitation for elderly patients with coronary heart disease: results from a randomised clinical trial. BMJ open. 2012;2(6). Epub 2012/12/21.
9. Beatty AL, Fukuoka Y, Whooley MA. Using mobile technology for cardiac rehabilitation: a review and framework for development and evaluation. Journal of the American Heart Association. 2013;2(6):e000568. Epub 2013/11/05.
10. Frederix I, Berger J, Hansen D, Van Driessche N, Bonne K, Alders T, et al. How to keep coronary artery disease patients active after the acute rehabilitation phase? The value of an internet-based telerehabilitation programme. ACTA CARDIOLOGICA. 2012;67(1):139-40.
11. Frederix I, Driessche NV, Hansen D, Berger J, Bonne K, Alders T, et al. Increasing the medium-term clinical benefits of hospital-based cardiac rehabilitation by physical activity telemonitoring in coronary artery disease patients. Eur J Prev Cardiol. 2013. Epub 2013/11/20.
12. Giordano A, Scalvini S, Zanelli E, Corra U, Longobardi GL, Ricci VA, et al. Multicenter randomised trial on home-based telemanagement to prevent hospital readmission of patients with chronic heart failure. International journal of cardiology. 2009;131(2):192-9. Epub 2008/01/29.
13. Durnin JV, Womersley J. Body fat assessed from total body density and its estimation from skinfold thickness: measurements on 481 men and women aged from 16 to 72 years. The British journal of nutrition. 1974;32(1):77-97. Epub 1974/07/01.
14. NVVC. Beslisboom Poliklinische Indicatiestelling Hartrevalidatie 2012. 2012; Available from: http://www.nvdietist.nl/temp/476873123/Beslisboom_Hartrevalidatie_2012_%28website%29.pdf.
15. Haddad M, Walters P, Phillips R, Tsakok J, Williams P, Mann A, et al. Detecting depression in patients with coronary heart disease: a diagnostic evaluation of the PHQ-9 and HADS-D in primary care, findings from the UPBEAT-UK study. PloS one. 2013;8(10):e78493. Epub 2013/10/17.

16.Spitzer RL, Kroenke K, Williams JB, Lowe B. A brief measure for assessing generalized anxiety disorder: the GAD-7. Archives of internal medicine. 2006;166(10):1092-7. Epub 2006/05/24.
17.McHorney CA, Ware JE, Jr., Lu JF, Sherbourne CD. The MOS 36-item Short-Form Health Survey (SF-36): III. Tests of data quality, scaling assumptions, and reliability across diverse patient groups. Medical care. 1994;32(1):40-66. Epub 1994/01/01.
18.McHorney CA, Ware JE, Jr., Raczek AE. The MOS 36-Item Short-Form Health Survey (SF-36): II. Psychometric and clinical tests of validity in measuring physical and mental health constructs. Medical care. 1993;31(3):247-63. Epub 1993/03/01.
19.Ware JE, Jr., Sherbourne CD. The MOS 36-item short-form health survey (SF-36). I. Conceptual framework and item selection. Medical care. 1992;30(6):473-83. Epub 1992/06/11.
20. Sofi, F, Cesari F, Abbate, R, Franco Gensini, G, Casini, A. Adherence to Mediterranean diet and health status: meta-analysis. British Medical Journal. 2008; 337;a1344
21.Lindsay J, Jr., Pinnow EE, Pichard AD. Frequency of major adverse cardiac events within one month of coronary angioplasty: a useful measure of operator performance. Journal of the American College of Cardiology. 1999;34(7):1916-23. Epub 1999/12/10.

13. APPENDIX I: Schedule of Events

PROCEDURE / TEST	Enrolment Randomisation (± 7 days)	End of *m*CR program (T1:6 months) (± 14 days)	End of study (T2 : 12 months) (± 14 days)
**Patient eligibility**	✓		
**Patient Informed Consent**	✓		
**Patient characteristics:**			
- Demographic	✓		
- Physical	✓		
- Medical history	✓		
- Risk factors	✓		
- Medication	✓		
- Indication for CR	✓		
**Centre characteristic**			
- Mobile telemonitoring guidance	✓		
**Maximal ergometer exercise test**	✓[1]	✓[1]	✓
**12-Lead ECG**	✓[1]	✓[1]	✓
**Physical examination:**			
- Weight, Length, BMI, Lean body mass, blood pressure	✓[1]	✓	✓
**Clinical Laboratory test:**			
- Lipid profile, renal function and HbA1C	✓[1]	✓[1]	✓
**Questionnaires**			
- SF36v2, PHQ-9, GAD 7, Mediterannean Diet Score	✓	✓	✓
**Care utilisation Monitioring[2]**	✓	✓	✓
**(Serious) Adverse Events Monitoring[2]**	✓	✓	✓

1. Routine practice
2. Between T1 (6 months) and T2 (12 months) monitoring of care utilisation and registration of events is done by monthly telephone calls with the participant.

14. APPENDIX II: Site investigator names and contact data per centre

- **Isala Zwolle, The Netherlands**
  Name investigator: Dr. E.P. De Kluiver
  E-mail: e.p.de.kluiver@isala.nl
  Phone: +31 (0)3 84 243 073

- **Radboudumc Nijmegen, The Netherlands**
  Name investigator: Dr. M.A. Brouwer
  E-mail: marc.brouwer@radboudumc.nl
  Phone: +31 (0)24 361 9350

- **Bispebjerg University Hospital Copenhagen (part of Region Hovedstaden), Denmark**
  Name investigator: Prof. Dr. E. Prescott
  E-mail: Eva.irene.bossano.prescott@regionh.dk
  Phone: +45 2257 2614/+45 4026 2134

- **Servizo Galego de Saude, Spain**
  Name investigator: Prof. J.R. Gonzalez-Juanatey
  E-mail: jose.ramon.gonzalez.juanatey@sergas.es
  Phone: +34 981 950 791

- **University of Bern, Switzerland**
  Name investigator: Dr. M. Wilhelm
  E-mail: matthias.wilhelm@insel.ch
  Phone: +41 (0)31 632 8970

- **Assistance Publique – Hopitaux de Paris, France**
  Name investigator: Dr. M. Iliou
  E-mail: marie-christine.iliou@ccl.aphp.fr
  Phone: +33 (0)1 58 004 260

15. APPENDIX III: Declaration of Helsinki

WORLD MEDICAL ASSOCIATION DECLARATION OF HELSINKI

Ethical Principles for Medical Research Involving Human Subjects

Adopted by the 18th WMA General Assembly, Helsinki, Finland, June 1964, and amended by the:

29th WMA General Assembly, Tokyo, Japan, October 1975

35th WMA General Assembly, Venice, Italy, October 1983

41st WMA General Assembly, Hong Kong, September 1989

48th WMA General Assembly, Somerset West, Republic of South Africa, October 1996

52nd WMA General Assembly, Edinburgh, Scotland, October 2000

53rd WMA General Assembly, Washington, DC, USA, October 2002 (Note of Clarification added)

55th WMA General Assembly, Tokyo, Japan, October 2004 (Note of Clarification added)

59th WMA General Assembly, Seoul, Republic of Korea, October 2008

64th WMA General Assembly, Fortaleza, Brazil, October 2013

A. INTRODUCTION

1. The World Medical Association (WMA) has developed the Declaration of Helsinki as a statement of ethical principles for medical research involving human subjects, including research on identifiable human material and data.

The Declaration is intended to be read as a whole and each of its constituent paragraphs should be applied with consideration of all other relevant paragraphs.

2. Consistent with the mandate of the WMA, the Declaration is addressed primarily to physicians. The WMA encourages others who are involved in medical research involving human subjects to adopt these principles.

B. GENERAL PRINCIPLES

3. The Declaration of Geneva of the WMA binds the physician with the words, “The health of my patient will be my first consideration,” and the International Code of Medical Ethics declares that, “A physician shall act in the patient's best interest when providing medical care.”

4. It is the duty of the physician to promote and safeguard the health, well-being and rights of patients, including those who are involved in medical research. The physician's knowledge and conscience are dedicated to the fulfilment of this duty.

5. Medical progress is based on research that ultimately must include studies involving human subjects.

6. The primary purpose of medical research involving human subjects is to understand the causes, development and effects of diseases and improve preventive, diagnostic and therapeutic interventions (methods, procedures and treatments). Even the best proven interventions must be evaluated continually through research for their safety, effectiveness, efficiency, accessibility and quality.

7. Medical research is subject to ethical standards that promote and ensure respect for all human subjects and protect their health and rights.

8. While the primary purpose of medical research is to generate new knowledge, this goal can never take precedence over the rights and interests of individual research subjects.

9. It is the duty of physicians who are involved in medical research to protect the life, health, dignity, integrity, right to self-determination, privacy, and confidentiality of personal information of research subjects. The responsibility for the protection of research subjects must always rest with the physician or other health care professionals and never with the research subjects, even though they have given consent.

10. Physicians must consider the ethical, legal and regulatory norms and standards for research involving human subjects in their own countries as well as applicable international norms and standards. No national or international ethical, legal or regulatory requirement should reduce or eliminate any of the protections for research subjects set forth in this Declaration.

11. Medical research should be conducted in a manner that minimises possible harm to the environment.

12. Medical research involving human subjects must be conducted only by individuals with the appropriate ethics and scientific education, training and qualifications. Research on patients or healthy volunteers requires the supervision of a competent and appropriately qualified physician or other health care professional.

13. Groups that are underrepresented in medical research should be provided appropriate access to participation in research.

14. Physicians who combine medical research with medical care should involve their patients in research only to the extent that this is justified by its potential preventive, diagnostic or therapeutic value and if the physician has good reason to believe that participation in the research study will not adversely affect the health of the patients who serve as research subjects.

15. Appropriate compensation and treatment for subjects who are harmed as a result of participating in research must be ensured.

C. RISKS, BURDENS AND BENEFITS

16. In medical practice and in medical research, most interventions involve risks and burdens.

Medical research involving human subjects may only be conducted if the importance of the objective outweighs the risks and burdens to the research subjects.

17. All medical research involving human subjects must be preceded by careful assessment of predictable risks and burdens to the individuals and groups involved in the research in comparison with foreseeable benefits to them and to other individuals or groups affected by the condition under investigation.

Measures to minimise the risks must be implemented. The risks must be continuously monitored, assessed and documented by the researcher.

18. Physicians may not be involved in a research study involving human subjects unless they are confident that the risks have been adequately assessed and can be satisfactorily managed.

When the risks are found to outweigh the potential benefits or when there is conclusive proof of definitive outcomes, physicians must assess whether to continue, modify or immediately stop the study.

D. VULNERABLE GROUPS AND INDIVIDUALS

19. Some groups and individuals are particularly vulnerable and may have an increased likelihood of being wronged or of incurring additional harm.

All vulnerable groups and individuals should receive specifically considered protection.

20. Medical research with a vulnerable group is only justified if the research is responsive to the health needs or priorities of this group and the research cannot be carried out in a non-vulnerable group. In addition, this group should stand to benefit from the knowledge, practices or interventions that result from the research.

E. SCIENTIFIC REQUIREMENTS AND RESEARCH PROTOCOLS

21. Medical research involving human subjects must conform to generally accepted scientific principles, be based on a thorough knowledge of the scientific literature, other relevant sources of information, and adequate laboratory and, as appropriate, animal experimentation. The welfare of animals used for research must be respected.

22. The design and performance of each research study involving human subjects must be clearly described and justified in a research protocol.

The protocol should contain a statement of the ethical considerations involved and should indicate how the principles in this Declaration have been addressed. The protocol should include information regarding funding, sponsors, institutional affiliations, potential conflicts of interest, incentives for subjects and information regarding provisions for treating and/or

compensating subjects who are harmed as a consequence of participation in the research study.

In clinical trials, the protocol must also describe appropriate arrangements for post-trial provisions.

F. RESEARCH ETHICS COMMITTEES

23. The research protocol must be submitted for consideration, comment, guidance and approval to the concerned research ethics committee before the study begins. This committee must be transparent in its functioning, must be independent of the researcher, the sponsor and any other undue influence and must be duly qualified. It must take into consideration the laws and regulations of the country or countries in which the research is to be performed as well as applicable international norms and standards but these must not be allowed to reduce or eliminate any of the protections for research subjects set forth in this Declaration.

The committee must have the right to monitor ongoing studies. The researcher must provide monitoring information to the committee, especially information about any serious adverse events. No amendment to the protocol may be made without consideration and approval by the committee. After the end of the study, the researchers must submit a final report to the committee containing a summary of the study's findings and conclusions.

G. PRIVACY AND CONFIDENTIALITY

24. Every precaution must be taken to protect the privacy of research subjects and the confidentiality of their personal information

H. INFORMED CONSENT

25. Participation by individuals capable of giving informed consent as subjects in medical research must be voluntary. Although it may be appropriate to consult family members or community leaders, no individual capable of giving informed consent may be enrolled in a research study unless he or she freely agrees.

26. In medical research involving human subjects capable of giving informed consent, each potential subject must be adequately informed of the aims, methods, sources of funding, any possible conflicts of interest, institutional affiliations of the researcher, the anticipated benefits and potential risks of the study and the discomfort it may entail, post-study provisions and any other relevant aspects of the study. The potential subject must be informed of the right to refuse to participate in the study or to withdraw consent to participate at any time without reprisal. Special attention should be given to the specific information needs of individual potential subjects as well as to the methods used to deliver the information.

After ensuring that the potential subject has understood the information, the physician or another appropriately qualified individual must then seek the potential subject's freely-given

informed consent, preferably in writing. If the consent cannot be expressed in writing, the non-written consent must be formally documented and witnessed.

All medical research subjects should be given the option of being informed about the general outcome and results of the study.

27. When seeking informed consent for participation in a research study the physician must be particularly cautious if the potential subject is in a dependent relationship with the physician or may consent under duress. In such situations the informed consent must be sought by an appropriately qualified individual who is completely independent of this relationship.

28. For a potential research subject who is incapable of giving informed consent, the physician must seek informed consent from the legally authorised representative. These individuals must not be included in a research study that has no likelihood of benefit for them unless it is intended to promote the health of the group represented by the potential subject, the research cannot instead be performed with persons capable of providing informed consent, and the research entails only minimal risk and minimal burden.

29. When a potential research subject who is deemed incapable of giving informed consent is able to give assent to decisions about participation in research, the physician must seek that assent in addition to the consent of the legally authorised representative. The potential subject's dissent should be respected.

30. Research involving subjects who are physically or mentally incapable of giving consent, for example, unconscious patients, may be done only if the physical or mental condition that prevents giving informed consent is a necessary characteristic of the research group. In such circumstances the physician must seek informed consent from the legally authorised representative. If no such representative is available and if the research cannot be delayed, the study may proceed without informed consent provided that the specific reasons for involving subjects with a condition that renders them unable to give informed consent have been stated in the research protocol and the study has been approved by a research ethics committee. Consent to remain in the research must be obtained as soon as possible from the subject or a legally authorised representative.

31. The physician must fully inform the patient which aspects of their care are related to the research. The refusal of a patient to participate in a study or the patient's decision to withdraw from the study must never adversely affect the patient-physician relationship.

32. For medical research using identifiable human material or data, such as research on material or data contained in biobanks or similar repositories, physicians must seek informed consent for its collection, storage and/or reuse. There may be exceptional situations where consent would be impossible or impracticable to obtain for such research. In such situations the research may be done only after consideration and approval of a research ethics committee.

I. USE OF PLACEBO

33. The benefits, risks, burdens and effectiveness of a new intervention must be tested against those of the best proven intervention(s), except in the following circumstances:

Where no proven intervention exists, the use of placebo, or no intervention, is acceptable; or

Where for compelling and scientifically sound methodological reasons the use of any intervention less effective than the best proven one, the use of placebo, or no intervention is necessary to determine the efficacy or safety of an intervention

and the patients who receive any intervention less effective than the best proven one, placebo, or no intervention will not be subject to additional risks of serious or irreversible harm as a result of not receiving the best proven intervention.

Extreme care must be taken to avoid abuse of this option.

J. POST-TRIAL PROVISIONS

34. In advance of a clinical trial, sponsors, researchers and host country governments should make provisions for post-trial access for all participants who still need an intervention identified as beneficial in the trial. This information must also be disclosed to participants during the informed consent process.

K.RESEARCH REGISTRATION AND PUBLICATION AND DISSEMINATION OF RESULTS

35. Every research study involving human subjects must be registered in a publicly accessible database before recruitment of the first subject.

36. Researchers, authors, sponsors, editors and publishers all have ethical obligations with regard to the publication and dissemination of the results of research. Researchers have a duty to make publicly available the results of their research on human subjects and are accountable for the completeness and accuracy of their reports. All parties should adhere to accepted guidelines for ethical reporting. Negative and inconclusive as well as positive results must be published or otherwise made publicly available. Sources of funding, institutional affiliations and conflicts of interest must be declared in the publication. Reports of research not in accordance with the principles of this Declaration should not be accepted for publication

L. UNPROVEN INTERVENTIONS IN CLINICAL PRACTICE

37. In the treatment of an individual patient, where proven interventions do not exist or other known interventions have been ineffective, the physician, after seeking expert advice, with informed consent from the patient or a legally authorised representative, may use an unproven intervention if in the physician's judgement it offers hope of saving life, re-establishing health or alleviating suffering. This intervention should subsequently be made the object of research, designed to evaluate its safety and efficacy. In all cases, new information must be recorded and, where appropriate, made publicly available.

STATISTICAL ANALYSIS PLAN

Randomised Controlled Trial for an eHealth application in EUropean Cardiac Rehabilitation programmes in Elderly

EU-CaRE RCT

Assessment of the effects on sustainability and participation level of a mobile telemonitoring guided cardiac rehabilitation programme (*m*CR) and its cost effectiveness, in elderly patients in Europe

PROTOCOL TITLE '*Randomised Controlled Trial for an eHealth application in European Cardiac Rehabilitation programmes in Elderly; Assessment of the effects on sustainability and participation level of a mobile telemonitoring guided cardiac rehabilitation programme (mCR) and its cost effectiveness, in elderly patients in Europe'*

**Protocol ID**	NL52862.075.15
**Short title**	Mobile telemonitoring guided cardiac rehabilitation programme (*m*CR) in elderly patients in Europe
**Abbreviation of title**	EU-CaRE RCT
**Protocol version**<br>**Date of issue**	1.3<br>08 July 2016
**Study registration**	Nederlands Trial Register<br>NTR 5308
**SAP version**<br>**Date of issue**	1.0<br>05 July 2017

SIGNATURE PAGE – APPROVAL

AUTHORS

PARTNER	WP	NAME	FUNCTION	DATE	SIGNATURE
DIAGRAM	NA	E. Kolkman, MSc	Statistician		
DIAGRAM	NA	E. Nibbering, MSc	Head Data management		
REGIONH	2	Prof. dr. E. Prescott	PI / WP leader		
UBERN	4	Dr. P. Eser			
IHF/ZAR	5	Dr. S. Schneider	Statistician		
IHF/ZAR	5	A. Piotrowski, MSc	Health economist		
SERGAS	6	Dr. C. Peña Gil			

SC MEMBERS (*if not already mentioned as author*)

PARTNER	WP	NAME	FUNCTION	DATE	SIGNATURE
ISALA	1	Prof. dr. A.W.J. van 't Hof	WP leader		
ISALA	3	Dr. A.E. van der Velde	WP leader		
UBERN	4	Dr. M. Wilhelm	PI / WP leader		
IHF/ZAR	5	Prof. U. Zeymer	PI / WP leader		
SERGAS	6	Prof. J.R. Gonzalez-Juanatey	PI / WP leader		
APHP	7	Dr. M.C. Iliou	PI / WP leader		
AOP	NA	Prof. D. Ardissino	PI		
RAD	NA	Prof. dr. M.J. de Boer	SC member		
MOBIHEALTH	NA	Dr. E. P. de Kluiver	SC member		
DIAGRAM	NA	J. Klijn	SC member		

Table of Contents

Revision Control Log 6

Abbreviations 7

Definitions 8

1. Introduction 9

1.1 Objectives of the Study Statistical Analysis Plan 9

1.2 Scope 9

1.3 Study details 9

1.4 Design of the study 10

1.5 Inclusion and exclusion criteria 10

1.6 Study schedule 11

2. Study objectives 12

2.1 Primary objective 12

2.2 Secondary objective 12

2.3 Tertiary objective 12

2.4 Fourth objective 12

2.5 Fifth objective 12

2.6 Sixth objective 12

2.7 Seventh objective 12

2.8 Eighth objective 13

3. Sample size 14

4. General Considerations 15

4.1 Timing of analyses 15

4.2 Definition of analysis sets 15

4.2.1 Full analysis population 15

4.2.2 Per protocol analysis population 16

4.2.3 Safety population 16

4.3 Data review meeting 16

4.4 Interim analysis..16
4.5 Subject disposition...16
4.6 Summary of study data 17
4.6.1 Demographic and baseline variables 17
4.7 Missing data..18
4.8 Subgroups 18
5. Study objective 1..19
5.1 Efficacy analysis of primary study endpoint...19
5.2 Analysis secondary endpoints...20
6. Study objective 2..21
7. Study objective 3..22
8. Study objective 4..23
9. Study objective 5..24
10. Study objective 6 25
11. Study objective 7 26
12. Study objective 8 27
13. Study objective 9 28
13.1 Identifying variables influencing participation and analysing participation rates..28
13.1.1. Strategy 1 29
13.1.2. Strategy 2 29
13.1.3. Strategy 3 29
13.2 Identifying and analysing variables influencing compliance and adherence during the study period...31
14. Safety...33
15. Amendment on the statistical analysis plan..34
16. Conduct of statistical analysis 35
17. Reporting conventions 36
18. Summary of changes to the protocol...37

Revision Control Log

Version	Date	Significant Changes from Previous Authorized Version
1.0	05-07-2017	NA

Abbreviations

ACS	Acute coronary syndrome
AE	Adverse event
BIC	Bayesian information criterion
BMI	Body mass index
CABG	Coronary artery bypass grafting
CAD	Coronary artery disease
CCR	Costs for cardiac rehabilitation
CCS	Canadian Cardiovascular Society
CCU	Costs for care utilization
COPD	Chronic obstructive pulmonary disease
CPET	Cardiopulmonary exercise test
CR	Cardiac rehabilitation
CRTP	Cardiac resynchronization therapy pacemaker
CV	Cardiovascular
CVA	Cerebrovascular accident
CVD	Cardiovascular disease
FEV	Forced expiratory volume
GCP	Good clinical practice
HDL	High-density lipoprotein
HSD	Honestly significant difference
ICD	Implantable cardioverter-defibrillator
ICER	Incremental cost effectiveness ratios
LDL	Low-density lipoprotein
LVEF	Left ventricular ejection fraction
MACE	Major adverse cardiovascular events
MI	Myocardial infarction
MMRM	Mixed models repeated measures
NYHA	New York Heart Association
OR	Odd ratio
OSA	Obstructive sleep apnea
PCA	Principal component analysis
PCI	Percutaneous coronary intervention
RCT	Randomised controlled trial
RER	Respiratory exchange ratio
SAE	Serious adverse event
SAP	Statistical analysis plan
TAVI	Transcatheter aortic valve implantation
TC	Total costs
TIA	Transient ischemic attack

Definitions

Definitions are captured in a separate document “EU-CaRE & EU-CaRE RCT SAP definitions” with version 1.0; 05 July 2017.

1. Introduction

1.1 Objectives of the Study Statistical Analysis Plan

The objective of this statistical analysis plan (SAP) is to outline the necessary statistical techniques to evaluate the efficacy and safety parameters outlined in the protocol. The SAP describes in detail which statistical analyses will be conducted.
The study protocol is the primary source of the SAP. Amendments or updates of sponsor requirements may result in a new version of this document or an amendment to the document. Changes from the protocol, if any, are provided in section 3.

The work in this study will be performed according to the Standard Operating Procedures of the beneficiaries, in accordance with the principles of good clinical practice (GCP) and ICHE9.

1.2 Scope

This SAP is based on the final protocol version 1.3 dated July 8, 2016. This SAP covers all endpoints for EU-CaRE RCT.

1.3 Study details

The aim of this randomised study is to investigate whether a *mobile* telemonitoring guided CR (*m*CR) programme as alternative for a regular CR programme is an effective means to increase participation and adherence of elderly in a CR programme, and results in better sustained effects than in patients who do not follow the *m*CR programme. In addition the induced and avoided costs and revenues of the *m*CR programme will be analysed in order to determine the cost effectiveness of this novel programme in comparison to other CR programmes.

1.4 Design of the study

This study is an open prospective, investigator initiated multicentre randomised controlled trial. A total of 238 cardiac patients (divided over six participating centres) suitable for CR (after approval cardiologist) and who do not volunteer to participate in the regular CR programme will be included in this interventional study and randomly assigned by an algorithm to one of the 2 study groups, i.e. the 6 months of *m*CR programme, or 6 months of no *m*CR programme. Both groups have an additional 6 months of follow-up after the first period without any intervention. Measurements in both groups will be performed at T0 (baseline), T1 (6 months follow up), and T2 (12 months follow up).

1.5 Inclusion and exclusion criteria

Inclusion criteria: Patients of 65 years or older who are a candidate for CR and non-voluntary to participate in the regular CR programme and signed a written consent and meet one of the following criteria within 3 months prior to start of the CR programme: 1) Patients with an acute coronary syndrome (ACS), including myocardial infarction (MI) and/or revascularisation, 2) Patients that underwent a percutaneous coronary intervention (PCI), 3) Patients that received coronary artery bypass grafting (CABG), 4) Patients who were treated surgically or percutaneously for valvular heart disease (including transcatheter aortic valve implantation (TAVI)), 5) Patients with a stable angina with documented significant coronary artery disease (CAD) (defined by standard non-invasive or invasive methods).

Exclusion criteria: Patients with a contraindication to CR, mental impairment leading to inability to cooperate, severe impaired ability to exercise, signs of severe cardiac ischemia and/or a positive exercise testing on severe cardiac ischemia, insufficient knowledge of the native language, no access, availability or insufficient knowledge of a computer with internet and an implanted cardiac device (pacemaker or implantable cardioverter-defibrillator (ICD)).

1.6 Study schedule

All patients will have a follow-up after 6 months and at 12 months after the randomisation. Measurements will be performed at T0 (baseline), T1 (6 months after randomisation), and T2 (12 months after randomisation), see figure 1.

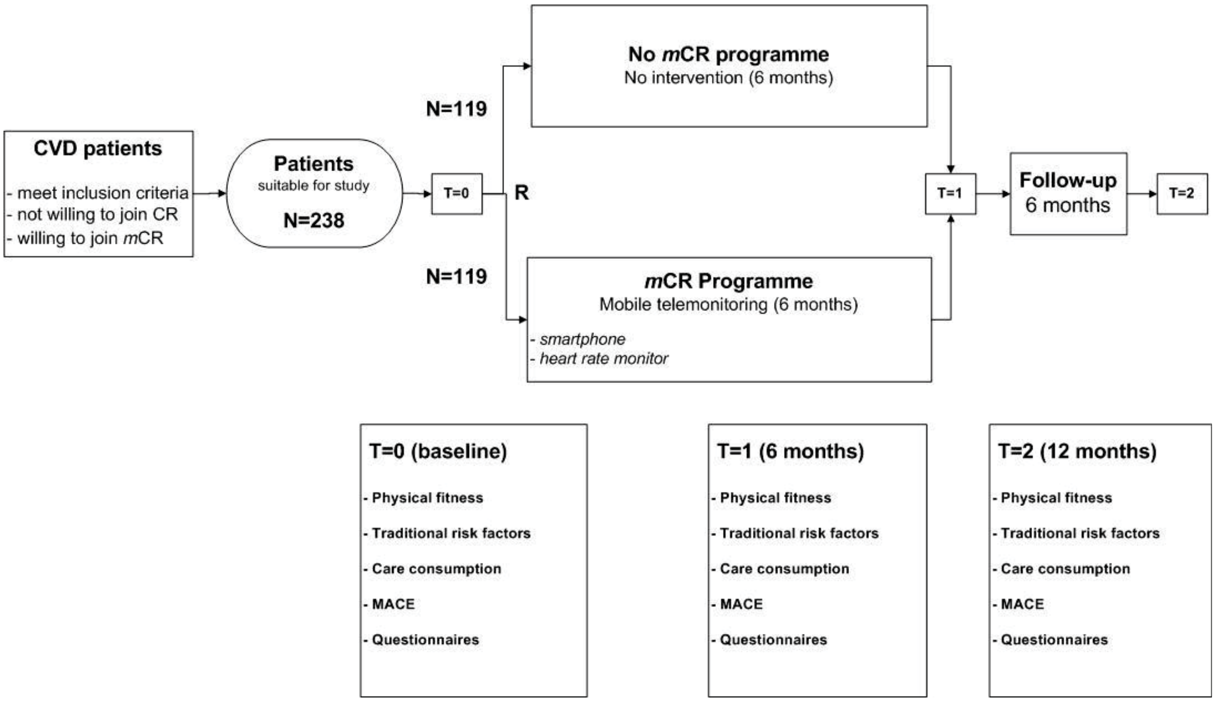

Figure 1. Study design EU-CaRE RCT

2. Study objectives

The randomised study has eight objectives.

2.1 Primary objective

Joining the *m*CR programme results in a smaller loss or greater gain in VO_{2peak} in cardiac patients between T1-T0 and T2-T0 than not joining the *m*CR programme.

2.2 Secondary objective

Joining the *m*CR programme results in smaller loss or greater gain in VO_{2peak} in cardiac patients between T2 and T1 than not joining the *m*CR programme.

2.3 Tertiary objective

Joining the *m*CR programme results in improved traditional risk profile (i.e. lipid profile, HbA1C, lean body mass, blood pressure) in cardiac patients in comparison to not joining the *m*CR programme after 6 (T1) and 12 months (T2).

2.4 Fourth objective

The overall occurrence of MACE (cardiovascular mortality, all-cause mortality, near sudden cardiac death, ACS, CV intervention/surgery, CV hospital admission, CV emergency visit) in the intervention group is lower than in the control group after T1 and T2.

2.5 Fifth objective

Joining the *m*CR programme results in an improved general health (e.g. quality of life (SF-36v2: Physical and Mental Component Summary Score), level of depression (PHQ-9), level of anxiety (GAD-7) and dietary pattern (Mediterranean Diet Score)) in comparison to not joining the *m*CR programme at T1 and T2 in cardiac patients.

2.6 Sixth objective

Utilisation of care in the intervention group is lower than in the control group throughout the study period.

2.7 Seventh objective

The costs of care utilisation in the intervention group are lower than in the control group throughout the study period.

2.8 Eighth objective

Cardiac patients randomised to the *m*CR programme comply with the use of the smartphone (use of the smartphone on at least 4 days per week with a minimum of 30 minutes of activity per day with a total registration of 150 minutes of activity per week during 21 weeks) during the first 6 months of the study.

3. Sample size

In order to assess the effects on sustained physical fitness after participation in the *m*CR programme it was determined that an inclusion of 119 patients per group would be sufficient to examine the aim of this study. This is based on the expected difference in increase in VO_{2peak} level of 3.0 ml/kg/min at 6 months between the control and intervention group with an estimated standard deviation of 5 ml/kg/min in the intervention group and 6 ml/kg/min in the control group. Assuming 80% power and 5% two-sided significance level (α=0.05), the sample size (n) required to achieve a probability of 80% of detecting a difference in an increase VO_{2peak} level between two independent groups is n=55 per randomisation group. Since patients from different centres (countries) will be pooled in the analysis, we will apply an adjustment for dependency of the observations to calculate the sample size, based on an estimated intraclass correlation coefficient (ICC) for the study centres. The ICC is the proportion of the total variance which is between the centers rather than within centres. An ICC of 0.05 is used in the sample size calculations. After adjustment for the ICC the sample size is N=83 per group. Based on 30% withdrawal we aim for a group size of n=119 per randomisation group (n=238 in total). Six centres will participate in the RCT so we will aim at including approximately 40 patients per centre. As this is a cooperative trial, centres may recruit more patients than this recruitment goal. When one centre starts to dominate enrolment however, recruitment might need to be capped. The total sample size (n=238) would be sufficient to detect a clinically and physiologically relevant change in peak oxygen consumption, despite a 30% dropout.

4. General Considerations

4.1 Timing of analyses

The final analysis of primary objective will be performed when all subjects have completed T1 or dropped out prior to T1. Data should meet the cleaning and approving requirements and SAP should be finalised and approved.

The final analysis of other objectives will be performed when all subjects have completed T2 or dropped out prior to T2. Data should meet the cleaning and approving requirements.

The cleaning and approving requirements are

- all expected CRFs have been entered,
- all queries are resolved,
- all data are consistent,
- data is determined to be clean and
- the expected centre signatures have been applied

4.2 Definition of analysis sets

This section is designed to identify the characteristics needed for inclusion in particular populations used in the analysis.

4.2.1 Full analysis population

The main outcome analyses will be based on the full analysis population. The full analysis population consists of all randomised subjects who signed informed consent. Subjects will be analysed according to the intervention group they were randomised to (intention to treat), irrespective whether the subjects complied to the use of the smartphone according to protocol.

4.2.2 Per protocol analysis population

The per protocol population consists of all subjects who adhere to the major criteria in the protocol and who have a cardiopulmonary exercise test (CPET) test with sufficient quality of tests at T0, T1 and T2. For the intervention group this implies subjects who complied with the use of the smartphone (use of the smartphone on at least 4 days per week with a minimum of 30 minutes of activity per day with a total registration of 150 minutes of activity per week during 21 weeks) during the first 6 months of the study.

Sufficient quality of CPET is defined as follows: identical protocol used in tests to be compared, respiratory exchange ratio (RER) ≥1.1 (or, if RER <1, if delta between T0 and T1 and/or T2 ≤0.05), and test duration ≥4 min (excluding warm-up), no signs of mask leakage or other equipment failure by visual inspection of CPET data.

4.2.3 Safety population

The safety population is equal to the full analysis population (section 4.2.1).

4.3 Data review meeting

No formal data review meeting is planned. Prior to the start of the analysis each subject's inclusion or exclusion status with regard to both analysis populations will be assigned on the basis of the criteria described in section 4.2.

4.4 Interim analysis

No formal interim analysis is planned.

4.5 Subject disposition

The number of subjects randomised, and of those who completed each of T0, T1 and T2 will be presented together with the number of patients in each analysis set. Numbers will be depicted overall and by intervention group.

4.6 Summary of study data

All continuous variables will be summarised using the following descriptive statistics: n (non-missing sample size), mean, standard deviation, median, Q1, Q3, minimum and maximum. The frequency and percentages (based on the non-missing sample size) of observed levels will be reported for all categorical measures. In general, all data will be listed, sorted by intervention group and subject, and when appropriate by visit number.

4.6.1 Demographic and baseline variables

Demographic and baseline variables will be summarised by intervention group and overall for subjects in the full analysis population and per protocol population. Age (years) will be summarised as continuous data. Gender (male vs female), ethnicity (Caucasian (Caucasian, North African, Turkish, Other Middle East), Negroid, Oriental (Pakistan/Indian, Eastern Asia) or Other), highest educational attainment (university, secondary/high school, primary school), currently employed (yes, unemployed, sick leave, early retirement for health reasons, retired), currently involved in volunteer work (yes/no), form of living (alone/not alone), habitat (urban/rural) will be summarised as categorical data. Anthropometric parameters and vital signs will be summarized as continuous data in a table including height (cm), weight (kg), body mass index (kg/m2), waist size (cm), hip size (cm), waist/ hip ratio, sum of the 4 skinfolds (mm), % of body fat, and lean body mass (kg), and resting blood pressure (systolic and diastolic, mmHg). Index diagnosis, comorbidity, left ventricular ejection fraction (LVEF), time from index to start exercise program, baseline blood pressure, laboratory measurements and medication will also be summarised.

The summary statistics will be produced in accordance with section 4.6.

4.7 Missing data

All collected data will be used in the data analysis, also data from the patient who discontinue the study until the time of study discontinuation. Missing data will be reported overall and by intervention and centre. We will describe patterns in missing data. We will analyse whether missing data are systematically different between both groups (*m*CR and control group) or whether they are related to baseline values or values on earlier time points. In case that the occurrence of missing values is systematically different between groups, or is related to other variables, multiple imputation methods will be used to calculate parameter estimates for sensitivity analysis.

4.8 Subgroups

The following important demographic or baseline value defined subgroups are to be analysed for different effects.

- Age
- Gender
- Index event: CAD versus VHR
- Educational attainment: low versus medium/high
- Per protocol: yes versus no

Subgroup analyses will be exploratory.

5. Study objective 1

Joining the *m*CR programme results in a lesser loss or greater gain in VO_{2peak} in cardiac patients between T1-T0 and T2-T0 than not joining the *m*CR programme. Primary study endpoint is physical fitness.

5.1 Efficacy analysis of primary study endpoint

The primary efficacy analysis will compare the change in peak oxygen uptake (VO2peak/kg) from baseline (T0) to the 6 months follow up (T1) between the intervention and control group in the full analysis set.

VO2peak/kg will be derived from CPET. Approximated VO2peak will be calculated from 6MWT as described under the definitions (document "EU-CaRE & EU-CaRE RCT SAP definitions").

The primary endpoint is the change in VO_{2peak} level between baseline (T0) and at 6 months (T1). It will be tested whether this change is different in the *m*CR and the control group by means of linear mixed models with the intervention as a fixed effect. The effect of centre on the outcome is modelled with a random effect. In the analyses we will adjust for the baseline value of VO_{2peak}. The null hypothesis is that there is no difference in mean change in VO_{2peak} level between the randomised groups. The alternative hypothesis is that the mean change in VO_{2peak} level differs between the randomised groups.

A covariance structure will be selected from several plausible structures. The covariance matrix resulting in the lowest Bayesian information criterion (BIC) fit statistic will be chosen.

To assess, if the underlying assumptions behind the mixed-effects model analysis are fulfilled, we will investigate normal quantile plots of residuals, standardized residuals, and random effects.

The linear mixed-effects model approach handles missing data appropriately and therefore multiple imputation or other methods to handle missing data in regard to the primary outcome will not be used. If necessary, the outcome variable will be transformed to obtain a normal distribution.

In case the groups differ on baseline values or other possibly confounding variables, the difference in change in VO_{2peak} level between baseline and 6 months between the intervention groups will be tested by means of linear mixed models analysis with centre as a random effect in which we will control for relevant confounders by adding covariates to the model. We will conclude a statistical significant difference in change in VO_{2peak} level between the *m*CR and control group when the two-sided p-value of the test statistic after adjustment for confounders is less than 0.05.

All analyses of PP will be in parallel to the description of the above.

5.2 Analysis secondary endpoints

The secondary endpoint for objective 1 is the change in peak oxygen uptake (VO2peak kg) from baseline (T0) to the 12 months follow up (T2) between the intervention groups in the full analysis set.

Differences between intervention groups in physical fitness from baseline (T0) to the 12 months follow up (T2) will be analysed in similar linear mixed model approach described in section 5.1.

All analyses of PP will be in parallel to the description of the above.

6. Study objective 2

Joining the *m*CR programme results in a smaller loss or greater gain in VO_{2peak} in cardiac patients between T2 and T1 than not joining the *m*CR programme.

Outcome is change in VO_{2peak} (T2-T1). Differences between intervention groups in VO_{2peak} will be analysed in similar linear mixed model like the primary outcome. It will be tested whether change in VO_{2peak} (T2 – T1) is different in the *m*CR and the control group by means of linear mixed models with the intervention as a fixed effect. The effect of centre on the outcome is modelled with a random effect. In the analyses we will adjust for the T1 value of VO_{2peak}. The null hypothesis is that there is no difference in mean change in VO_{2peak} level between the randomised groups. The alternative hypothesis is that the mean change in VO_{2peak} level differs between the randomised groups.

A covariance structure will be selected from several plausible structures. The covariance matrix resulting in the lowest BIC fit statistic will be chosen.
To assess, if the underlying assumptions behind the mixed-effects model analysis are fulfilled, we will investigate normal quantile plots of residuals, standardized residuals, and random effects.

Additionally, we will perform a mixed models repeated measures (MMRM) analysis with intervention effect and time points as fixed effect and centre as random effect and baseline value as covariate. When necessary the outcome variables will be transformed to obtain a normal distribution. The null hypothesis is that the outcome under study is the same at all-time points (T0, T1 and T2). The alternative hypothesis is that the outcome under study at least one of the time points differs from the others. A covariance structure will be selected from several plausible structures. The covariance matrix resulting in the lowest BIC fit statistic will be chosen. We will test for the interaction between intervention group and time in order to investigate whether the development of study parameters over time differs between the interventions. A two-sided p-value of less than 0.05 for will be considered to be statistically significant. If the results of the MMRM analysis are statistically significant, we will run post hoc tests to highlight where these differences occur, adjusting the p value for multiple comparisons using the Tukey procedure.

7. Study objective 3

Joining the *m*CR programme results in improved traditional risk profile (i.e. lipid profile, HbA1C, lean body mass, blood pressure) in cardiac patients in comparison to not joining the *m*CR programme after 6 (T1) and 12 months (T2).

Outcomes

Cardiovascular risk factor control:

- Changes in lipid profile (T1-T2)
- Changes in HbA1C (T1-T2)
- Changes in lean body mass (T1-T2)
- Changes in blood pressure (T1-T2)

The differences between the randomisation groups on the secondary continuous outcomes will follow the same approach as described in section 6 Study objective 2.

All analyses of PP will be in parallel to the description of the above.

8. Study objective 4

The overall occurrence of MACE (cardiovascular mortality, all-cause mortality, near sudden cardiac death, ACS, CV intervention/surgery, CV hospital admission, CV emergency visit) in the intervention group is lower than in the control group at T1 and T2.

The cumulative incidences of the composite endpoint MACE and the elements from which it is constituted will be presented by group. We will compute the probability of survival free from MACE past given time points (6 months and 12 months) and the survival function will be plotted by intervention group in Kaplan-Meier survival curves. The Kaplan Meier curves will indicate the occurrence of MACE during 12 months of follow-up. Patients who do not experience an event or are lost to follow-up without an event are censored. We will test for differences in hazard rates between the *m*CR and control group using Cox regression analysis allowing the baseline hazard functions to differ between centres.

9. Study objective 5

Joining the *m*CR programme results in an improved general health (e.g. quality of life (SF-36v2: Physical and Mental Component Summary Score), level of depression (PHQ-9), level of anxiety (GAD-7) and dietary pattern (Mediterranean Diet Score)) in comparison to not joining the *m*CR programme after T1 and T2 in cardiac patients.

Outcomes

General Health:

- Changes in Quality of Life: SF-36v2, difference in Physical Component Summary Score and Mental Component Summary Score and 8 domains (T1-T0), (T2-T0)
- Changes in depression score assessed by PHQ-9 questionnaire (T1-T0), (T2-T0)
- Changes in anxiety score assessed by GAD-7 questionnaire (T1-T0), (T2-T0)
- Difference in dietary pattern (reflected by total score) assessed by Mediterranean Diet Score (T1-T0), (T2-T0)

The differences between the randomisation groups on the general health continuous outcomes will follow the same approach as described in section 5 Study objective 1.

10. Study objective 6

Utilisation of care in the intervention group is lower than in the control group throughout the study period.

Differences between intervention groups in utilisation of medical care will be tested by linear mixed models with centre as random effect for continuous outcome or mixed models for logistic regression for dichotomous outcomes. When necessary continuous outcome variables will be transformed to obtain a normal distribution.

11. Study objective 7

To investigate the thesis that the costs of care utilisation in the intervention group are lower than in the control group throughout the study period.

Health care cost estimation:

Direct costs for mobile cardiac rehabilitation will be assigned for the intervention group (with telemonitoring guidance), but not for the control group (no intervention). Care utilization based on activities (clinical admission days, emergency and outpatient clinic visits, GP visits for cardiac (related) complaints or issues, radiology/cardio physiology/nuclear and laboratory tests, and cardiac interventions) will be registered at T1 and T2 and collected by monthly telephone calls with the participants between T1 and T2. A price will be assigned to each service and summed up for each patient individually:

	**Mobile CR**	**control**
**Costs for CR**	Yes	No
**Costs for care utilization**	Yes	Yes

Therefore, total costs (= TC) per patient comprise:

1. Costs for telemonitoring guidance (= CCR, only intervention group)
2. Sum of costs for medical services used outside CR (care utilization, = CCU).

Linear mixed models with centre as random effect will test differences between intervention and control group regarding total costs of care utilisation (see also section 5.1).

Additionally, incremental cost effectiveness ratios (ICER) defined as

$$\frac{Total\ costs\ mobile\ CR\ - Total\ costs\ control\ group}{Outcome\ mobile\ CR\ - Outcome\ control\ group}$$

will be estimated and a 95% confidence interval will be calculated. Outcome refers especially to the change of peak VO2 and Quality of Life (measured by Physical Component Summary Score and Mental Component Summary Score) during the follow-up period.

12. Study objective 8

Cardiac patients randomised to the *m*CR programme comply with the use of the smartphone (use of the smartphone on at least 4 days per week with a minimum of 30 minutes of activity per day with a total registration of 150 minutes of activity per week during 21 weeks) during the first 6 months of the study.

Descriptive statistics will be utilised to obtain insight in the use of the smartphone during the first six months of the study. Listed below are the parameters that will be determined.

- Total time (in hours) use of physical activity monitor
- Total time (in hours) use of physical activity monitor with light to moderate intensity
- Total time (in hours) use of physical activity monitor with moderate to high intensity
- Total time (in hours) use of physical activity monitor with high to extreme intensity
- Number of weeks physical activity monitor is used
- Total number of sessions
- Percentage of weeks subject has reached the exercise goal
- Percentage of patients that comply to the exercise goal

Parameters will be summarised in accordance with section 4.6. Summaries will be reported overall and by group.

13. Study objective 9

One of the main challenges for CR programmes, especially in elderly is to improve participation, adherence and compliance. A significant number of patients refuse or do not follow CR programmes. In this objective the variables (social, psychological, motivational and medical) are identified that could influence participation and adherence. Especially interesting is if new services guide by eHealth solutions (*m*CR) can improve adherence rates as well as participation rates.

Data from this study is analysed to define the preconditions for sustainable participation, adherence and compliance. Not participating will be defined by screening failure EU-CaRE RCT (see "EU-CaRE and EU-CaRE RCT SAP definitions" document).

The participation rates in all CVD patients that fulfil the inclusion and none of the exclusion criteria, mentioned in section 1.5, and participate in the randomised controlled trial (RCT) are studied. Compliance and adherence, analysing the drop-outs in the RCT is studied. The methodology and statistical methods will be the same used in the other objectives in addition to other suitable approaches related to each specific objective.

For a more homogenous analysis we will study participation in CAD (inclusion criteria 1,2 and 3 of section 1.5) and in each inclusion criteria group.

13.1 Identifying variables influencing participation and analysing participation rates

Social, psychological, motivational, contextual and medical variables will be incorporated. All registered variables will be taken into account, predictors of participation and non-participation will be identified with multivariable models (multiple logistic regression analysis). Differences between European regions, different programmes and ages will be studied.

Statistical modelling of participation (to know the relative weight of different variables and how they can be combined to maximize the real interest on patients). This would also imply identifying the profiles or targets in which the pervasiveness of the program can be higher and, on the contrary, those targets or situations in which resistances or barriers may be greater.

Three complementary strategies are proposed to reach the objective 9:

13.1.1. Strategy 1

A literature review searching the possible explanatory or moderator variables of participation, from the strictly medical ones, to sociodemographic ones, united to the psychosocial variables (like the awareness of own health status, the locus of control, expectations, motivation, mood state, self-esteem, family support, quality of life). See *Ruano-Ravina A et al. Participation and adherence to cardiac rehabilitation programs. A systematic review. Int J Cardiol. 2016;223:436-443. doi: 10.1016/j.ijcard.2016.08.120*]

13.1.2. Strategy 2

A qualitative study, with Focus Groups and individual interviews to current users as well as potential users, health professionals (from primary care and cardiology services). It allows us not only to know the attitudes and perceptions about the program, but also to identify participation resistances or barriers. At the same time it provides us possible keys in terms of communication which should be used to increase the value of the program, making it more attractive for the potential users.

13.1.3. Strategy 3

A quantitative study. We propose to estimate the probability of participation in the RCT according to different variables collected in participants and non-participants. We will perform firstly a descriptive univariate analysis of the included variables followed by logistic regression models. Bivariate analysis will compare the characteristics of participants in CR programs with non-participants. To compare these characteristics we will use Pearson's ji squared test or Mann-Whitney's U as appropriate.

We will perform different logistic regression models were the dependent variable will be participation or not. As independent variables we are going to use information collected in screened participants and grouped in different areas (see potential predictors for participation). We aim to include the maximum number of variables to try to explain participation and identify which characteristics are more tightly linked with participation. We will not use automatic procedures for the inclusion of variables in the models. The results will be expressed as odds ratios (OR) with 95% confidence interval (95%CI). For all models, we will calculate the Hosmer-Lemeshow calibration test and the C index. We

will compare the performance of the models using receiver operating characteristic (ROC) curves. We will select the model with the highest C index but including a low number of variables (looking for a parsimonious model explaining participation with the lowest number of covariates). This will enable the identification of patients more reluctant to participate and perform a more active information on them.

Potential predictors for participation

- Reason for not accepting cardiac rehabilitation (see "EU-CaRE & EU-CaRE RCT SAP definitions")

<u>Socio-economic characteristics:</u>

- Regular CR programme totally covered by health insurance

Socio-demographic variables:

- Country
- Geographical region
- Age
- Gender
- Ethnicity
- Educational attainment
- Employment status
- Currently involved in volunteer work (Yes, No)
- Co-habitation
- CR programme covered by health insurance
- Specify Totally covered Partially covered
- Urban/rural
- Number of kilometres from home to rehabilitation centre
- Number of kilometres from home to clinic
- Number of kilometres from home to general practitioner
- Index event
- Time lapse from index event to T0 CPET
- Symptoms (CCS & NYHA class)
- Medication use T0
- CVD risk factors prior to index event (smoker, diabetes mellitus type I/II, hypercholesterolemia, hypertension, family history CVD, physical activity prior to

index event, number of alcoholic units per week/number of days with alcoholic consumption)

- Co-morbidity (Previous ACS, PTCA/PCI or CABG, chronic heart failure, valvular heart disease, chronic/persistent atrial fibrillation, COPD, OSA, TIA/CVA, rheumatoid arthritis, nephropathy, peripheral artery disease, current depression)

As an exploratory analysis we will perform a separate analysis by recruitment centre to detect possible differences in models explaining participation according to different patient's characteristics.

Additionally the following analyses will be considered

- Structural Equation Models (3 Dependent Variables: interest and confidence in the program and expectations fulfilment).
- Cluster Analysis to identify and characterize different profiles of potential users and link them to the interest level in the program.

13.2 Identifying and analysing variables influencing compliance and adherence during the study period

Qualitatively the incidence of the different causes of drop-out will be analysed, globally and per centre. We will differentiate between cardiac and medical reason for drop-out and non-medical reasons.
Analysing the differences in compliance and adherence of patients treated with mHealth solutions (intervention) during the study period.

Statistical modelling of adherence and compliance to maximize the continuation of patients on the program and even make possible that guidelines and changes established would be maintained, so that the benefits of the program would be really durable.

The following information will be used for the statistical modelling.

End of *m*CR programme (T1) data:

- Censoring variable: drop-out *m*CR programme
- Response variable: Follow-up time (months or weeks)
- Predictor variables: in addition to general input data, medical data about the patient evolution can be used.

Data Analysis:

- Survival Analysis (Kaplan-Meier tables with descriptive statistics, Log Rank Test to compare survival by groups and Cox Regression to model survival and calculate Hazard Ratios)
- Logistic Regression (to explain drop-out and compliance)

Potential predictors

Variables measured T0

- Adverse event (AE)/serious adverse event (SAE)
- Physical parameters: BMI, lean body mass
- Heart Rhythm
- Forced expiratory volume (FEV)1 L*min
- Lab (LDL, HDL, Total cholesterol, Triglyceride, HbA1C, Creatinine, CRP, Hb)
- Physical activity prior to index event
- Symptoms (CCS & NYHA class)
- Medication use

Variables measured at T1

- AE/SAE
- Drop-out *m*CR programme
- Reason drop-out of CR programme
- Drop-out study
- Reason drop-out of study
- Attendance to multidisciplinary sessions
- Medication use

Additionally the following analyses will be considered:

- Structural Equation Models (to explain satisfaction and fulfilment of expectations)
- Cluster Analysis (to identify and characterize different profiles of users and link them to the program adherence)

14. Safety

The safety population will be used to describe the safety. Safety data will be coded according to MedDRA PT and will be summarised in tables by intervention group, centre and overall.

Adverse events

Deaths and SAEs will be listed on patient level by intervention group.
When calculating the incidence of AEs, or any sub classification thereof by intervention, time period, severity, etc. each subject will only be counted once and any repetitions of AEs will be ignored; the denominator will be the total population size.

15. Amendment on the statistical analysis plan

In case of an amendment of the protocol or for other reasons the SAP may be amended accordingly. All amendments will be logged and registered.

Only SAP and "EU-CaRE & EU-CaRE RCT SAP definitions" document with version 1.0 will be approved with a signature of all "authors" and "SC members" as registered on the "signature page" of this SAP.
All *substantial* changes of both documents after initial approved version 1.0 need approval from the "authors". A substantial change is marked via a full upgrade of the version number (e.g. 2.0 to 3.0 or 2.1 to 3.1) of the related document.
All *non-substantial* changes after initial approved version 1.0 only need approval from the "authors" of coordinating partner Diagram. A non-substantial change is marked via a partly upgrade of the version number (e.g. 2.0 to 2.1 or 3.1 to 3.2) of the related document.

16. Conduct of statistical analysis

All statistical calculations will be performed using SAS version 9.3 or higher, SPSS version 19 and R version 3.3.1., R Core Team (2016). Statistical analysis will be performed by the beneficiaries Bern, Santiago, Ludwigshafen, Copenhagen and CRO Diagram. Diagram will have an overview of all analyses that will be performed.
Diagram, as reviewing party, will independently reproduce the primary analyses and summary statistics table X, Y, Z. Diagram will explicitly check the code producing tables A, B & C (selected at random) as well as other pieces of code as desired. Each beneficiary is responsible for quality control of all macros and programs used during the analysis.

17. Reporting conventions

The following reporting conventions will be applied. P-values ≥ 0.001 will be reported to 3 decimal places. P values less than 0.001 will be reported as "< 0.001". The mean, standard deviation, and any statistics other than quantiles, will be reported to one decimal place greater than the original data. Quantiles, such as median, or minimum and maximum will use the same number of decimal places as the original data. Estimated parameters, not on the same scale as raw observations (e.g. regression coefficients) will be reported to 3 significant digits.

18. Summary of changes to the protocol

A per protocol analysis set is added to the SAP. Analysis for primary and secondary endpoints will be repeated for the per protocol population. In the per protocol analysis population we will take into account the subjects who adhere to the protocol in terms of subjects who adhere to the major criteria in the protocol and who have a cardiopulmonary exercise test (CPET) test with sufficient quality of tests at T0, T1 and T2. For the intervention group this implies subjects who complied with the use of the smartphone (use of the smartphone on at least 4 days per week with a minimum of 30 minutes of activity per day with a total registration of 150 minutes of activity per week during 21 weeks) during the first 6 months of the study. The per-protocol restricts the comparison of the treatments to the ideal patients, that is, those who adhered perfectly to the clinical trial instructions as stipulated in the protocol. A per-protocol analysis answers the questions as to whether the treatment works among those that comply, but it can never provide an unbiased assessment of the true effect since the decision to comply with a treatment is unlikely to occur at random. Both will be used as supportive analysis.
