# Supplementary material for: Effectiveness of Home-Based Mobile Guided Cardiac Rehabilitation as Alternative Strategy for Nonparticipation in Clinic-Based Cardiac Rehabilitation Among Elderly Patients in Europe: A Randomized Clinical Trial
Source: JAMA Cardiol. 2020 Oct 28;6(4):1–6. doi: 10.1001/jamacardio.2020.5218 (PMC7593879; doi:10.1001/jamacardio.2020.5218)
Supplement: Supplement 2. — eMethods. Design and Participants eFigure 1. Flowchart of Study Protocol With All Contact Moments and Measurements eFigure 2. Main Reasons for Not Participating in Cardiac Rehabilitation eTable 1. Baseline Characteristics eTable 2. Primary and Secondary Outcome Parameters at Baseline and 6- and 12-Month Follow-up eTable 3. Adverse Events Within Study Period [file jamacardiol-e205218-s002.pdf]

Supplementary Online Content

Snoek JA, Prescott EI, van der Velde AE, et al. Effectiveness of home-based mobile guided cardiac rehabilitation as alternative strategy for nonparticipation in clinic-based cardiac rehabilitation among elderly patients in Europe: a randomized clinical trial. *JAMA Cardiol*. Published online October 28, 2020. doi:10.1001/jamacardio.2020.5218

eMethods. Design and Participants
eFigure 1. Flowchart of Study Protocol With All Contact Moments and Measurements
eFigure 2. Main Reasons for Not Participating in Cardiac Rehabilitation
eTable 1. Baseline Characteristics
eTable 2. Primary and Secondary Outcome Parameters at Baseline and 6- and 12-Month Follow-up
eTable 3. Adverse Events Within Study Period

This supplementary material has been provided by the authors to give readers additional information about their work.

© 2020 Snoek JA et al. *JAMA Cardiology.*

eMethods. Design and Participants

Study design

The EUropean study on effectiveness and sustainability of current Cardiac Rehabilitation programs in the Elderly (EU-CaRE) RCT is an international multicenter parallel randomized clinical trial (RCT). The study was conducted in accordance with the declaration of Helsinki. Six European cardiac institutions across five European countries participated in the EU-CaRE RCT: Isala Heart Center (Netherlands), Region Hovedstaden (Denmark), Assistence Publique – Hôpitaux de Paris (France), Universität Bern (Switzerland), Servizo Galego de Saúde (Spain) and Radboud University Medical Center (Netherlands). The study protocol was approved by all local ethics committees and informed written consent was obtained from all participants. Details of the study methods have been published previously.[1]

Patients

Patients with recent (< 3 months) acute coronary syndrome, coronary revascularization, surgical or percutaneous treatment for valvular disease or documented CAD defined by standard non-invasive or invasive methods, living in the catchment area of the participating CR centers and declining participation in conventional CR were eligible for inclusion. Exclusion criteria were any contraindication to CR, mental impairment leading to inability to cooperate, severely impaired ability to exercise, signs of severe cardiac ischemia and/or positive exercise testing on cardiac ischemia, insufficient knowledge of the native language or an implanted cardiac device (CRT-P, ICD). Patients were recruited by a research assistant from the participating centers by screening all consecutive patients admitted to the participating centers.

© 2020 Snoek JA et al. *JAMA Cardiology.*

Randomization and blinding

Randomization was performed in fixed blocks of n=4, stratified by center, with a 1:1 ratio to the intervention group (*m*CR) or a control group without CR (CON) by means of a centralized computerized allocation system applying an algorithm that prevented the care providers or the investigators from predicting the outcome of the randomization process. Both patients and personnel were unaware of the group the patient would be allocated to at the time of performing the baseline cardiopulmonary exercise test (CPET). The nature of the intervention made it impossible to blind patients and personnel involved in the guidance in *m*CR, but researchers assessing primary outcome assessment were masked for group assignment.

Procedures

Intervention group

The intervention has been previously described.[1] In short, patients were given a 6-months *m*CR program followed by a 6-months follow-up period without telemonitoring and coaching. *m*CR involved a home-based CR program in which patients were equipped with a smartphone and connected heartrate belt. Patients were able to measure and record training mode, duration and intensity (determined by continuously recorded and simultaneously displayed heart rate). Subjects were instructed to exercise at a moderate intensity for at least 30 minutes at 5 days/week.[2] Moderate intensity was defined as the intensity above the first ventilatory threshold determined at baseline CPET.[3] They were contacted weekly by telephone for motivational interviewing the first month, every other week the second month and from then on monthly until completion of the *m*CR program at six months. All researchers involved in the present study received training on motivational interviewing during the initiation visit.[4] Physical activity patterns were discussed by telephone using a similar standardized mode of operation across study sites. After 6 months the smartphone and

© 2020 Snoek JA et al. *JAMA Cardiology.*

heart rate belt were handed in. In the subsequent six months, patients received no further coaching or feedback. An overview of study procedures is presented in Supplement figure 1.

Control group

Patients in the control group did not receive any form of CR but received locally defined standard of care during the 12-month study period including counselling on healthy exercise behavior but without any form of coaching and/or guidance to change habitual physical activity from the participating CR centers.

Outcomes

Patient assessment and measurements in both groups were performed at baseline, six and twelve months.

Physical fitness. Primary endpoint was the comparison of both groups with respect to changes in physical fitness from baseline to follow-up at six months. Physical fitness was defined as peak oxygen uptake (VO_{2peak}). All sites used the same standard operating procedures for calibration, instruction and measurements for CPET according to international guidelines.[5] Equipment varied between sites, but identical protocols were used in all tests within each patient. Patients started with a 3-minute warm-up at 5 Watt. Subsequently an individualized ramp protocol was performed in order to reach VO_{2peak} after 8-12 minutes. We aimed to surpass the first ventilatory threshold and a respiratory exchange ratio >1.1. Raw data of all tests were analyzed at the core lab (Bern) by an automated procedure on raw data files using MATLAB software (MathWorks® version 9.3, release R2017b, Natick Massachusetts, USA). Visual quality control was performed by one experienced operator (TM) and in case of doubtful quality by a second operator (MW). The highest 30s moving average was considered as VO_{2peak}. In case of mask intolerance, signs of mask leakage

© 2020 Snoek JA et al. *JAMA Cardiology.*

or short test duration (< 3 minutes) VO_{2peak} was calculated with a validated formula using maximal workload.[6] If patients were not able to perform a CPET on a bicycle or a treadmill, a six-minute walking test was executed to estimate VO_{2peak} using a validated formula.[7]

Biological markers. Standardized clinical chemical blood tests were performed to determine total cholesterol, low density lipoprotein (LDL) cholesterol, high density lipoprotein (HDL) cholesterol and HbA1c in local laboratories.

Physical activity. Self-reported PA was assessed using the following 2 questions: "How many days per week do you perform moderate (walking, carrying light loads, mopping/vacuuming, bicycling at regular pace or gardening) to vigorous (heavy lifting, digging, aerobics, running or fast bicycling) PA?" and "How many minutes per day do you perform moderate to vigorous PA?" The definition of self-reported habitual PA was considered the total number of days per week in which a minimum of 30 minutes of self-reported moderate to vigorous PA was registered.

Questionnaires. Quality of life was assessed with the second version of the 36-item short form health survey (SF-36v2)[8], anxiety with the general anxiety disorder questionnaire (GAD-7)[9] and depression with the patient health questionnaire (PHQ-9).[10]

Major adverse cardiovascular events (MACE). Events were registered and collected by monthly telephone calls with the participants in both groups throughout the study period and evaluation of patients' electronic medical files. MACE was defined as a composite of all-cause mortality, cardiovascular mortality, near sudden cardiac death, acute coronary syndrome and hospital admission for cardiovascular disease (CVD).

Statistical analysis

Sample size calculation was based on the expected difference in increase in VO_{2peak} of 3.0 $mL \cdot kg^{-1}min^{-1}$ between the control and intervention group from baseline to 6 months with an estimated SD of 5.0 in the intervention group and 6.0 in the control group.[11] Assuming 80% power and

© 2020 Snoek JA et al. *JAMA Cardiology.*

a 5% two-sided significance level (α=0.05), the sample size (*n*) required to achieve an 80% probability of detecting a difference in the increase between two independent groups was *n*=55 per randomization group. Since this was a multi-center study in different countries, we used an adjustment based on the estimated intraclass correlation coefficient (ICC) for the study centers to calculate sample size. An ICC of 0.05 was used. After adjustment for the ICC, sample size was *n*=83 per randomization group. Based on a 30% drop-out rate during follow-up, *n*=119 per group (*n*=238 in total) would be needed to recruit.

All parameters were analyzed according to the initial randomization. Distribution of the data was assessed with the Shapiro Wilk test. Continuous variables are presented by mean ± standard deviation (SD) and categorical variables by count and percentage. Analyses were performed using SAS 9.4 (SAS Institute Inc., Cary, NC, USA).

Time-dependent changes within groups were tested with paired sample t-tests. Primary outcome was assessed by a linear mixed model with change in VO_{2peak} (6 months - baseline) as response, the intervention (*m*CR *versus* CON) as a fixed effect, the effect of center as a random effect and adjustment for baseline value of VO_{2peak}. Secondary outcome parameters were likewise analyzed by a linear mixed model with adjustment for baseline values of the dependent variable. Two-sided $p<0.05$ was considered statistically significant.

A Clinical Event Committee (CEC) reviewed and adjudicated all clinical endpoint events.

© 2020 Snoek JA et al. *JAMA Cardiology.*

eFigure 1. Flowchart of Study Protocol With All Contact Moments and Measurements

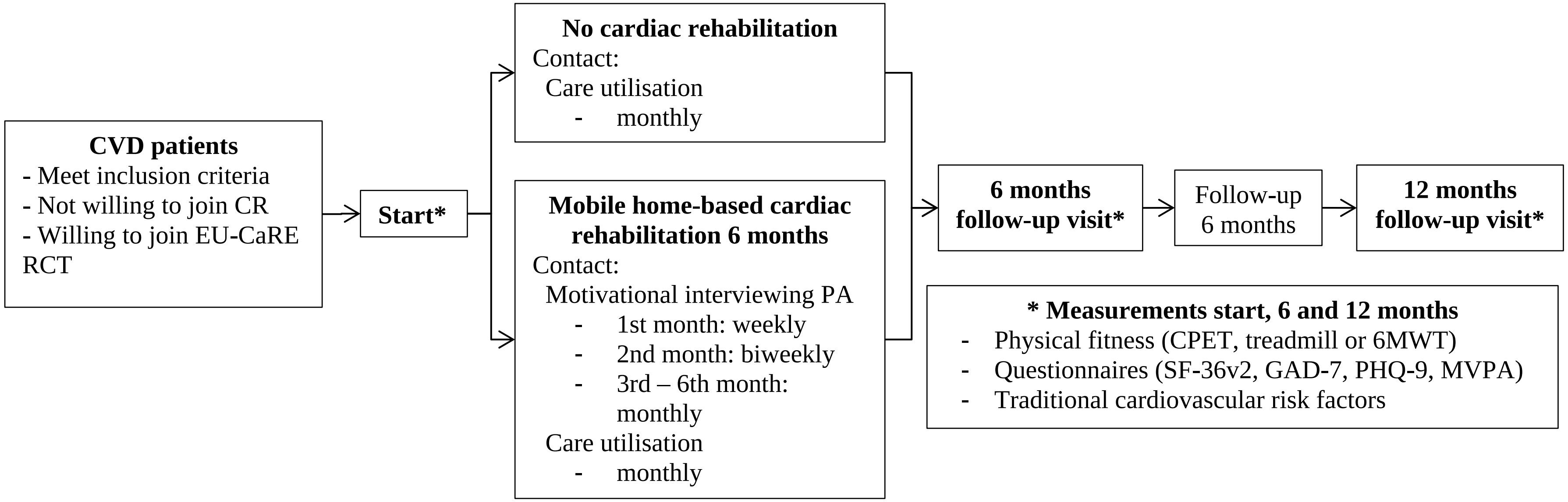

CR, cardiac rehabilitation; PA, physical activity; CPET, cardiopulmonary exercise test; 6MWT six minute walking test; SF36v2, short form 36 version 2; GAD-7, general anxiety disorder questionnaire; PHQ-9, patient health questionnaire; MVPA moderate to vigorous physical activity

© 2020 Snoek JA et al. *JAMA Cardiology.*

eFigure 2. Main Reasons for Not Participating in Cardiac Rehabilitation

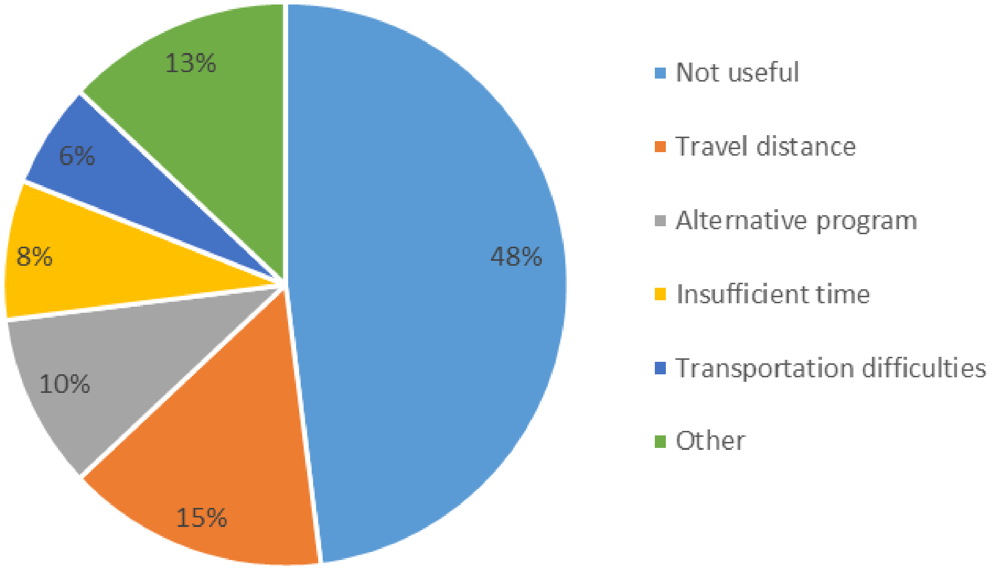

© 2020 Snoek JA et al. *JAMA Cardiology.*

eTable 1. Baseline Characteristics

Characteristic	Treatment group	
	**MCR (n = 89)**	**Control (n = 90)**
Age, mean (SD), y	72.4 (5.4)	73.6 (5.5)
Female, No. (%)	20 (22)	14 (16)
White	88 (99)	89 (99)
BMI, mean (SD)	27.6 (4.0)	27.0 (4.2)
Blood pressure, mean (SD), mm Hg		
Systolic	134 (19)	130 (18)
Diastolic	78 (11)	76 (12)
Waist size, mean (SD), cm	100 (10)	99 (10)
Total cholesterol level, mean (SD), mg/dL	158 (4)	158 (4)
LDL cholesterol level, mean (SD), mg/dL	85 (31)	85 (27)
HbA_{1c} level, mean (SD), mmol/mol	42.6 (9.4)	40.0 (5.7)
Distance from home to cardiac rehabilitation center, mean (SD), km	21 (22)	21 (24)
Cardiovascular risk factors, No. (%)		
Smoker	3 (3)	5 (6)
Previous smoker	41 (46)	43 (48)
Hypertension	73 (82)	60 (67)
Diabetes	23 (26)	15 (17)
Inactivity	41 (46)	45 (50)
Hypercholesterolemia	74 (83)	71 (79)
Family history	30 (34)	32 (36)
Index intervention, No. (%)		
PCI	63 (71)	65 (72)
CABG	11 (12)	8 (9)
Valve replacement	1 (1)	3 (3)
None	14 (16)	14 (16)
Medication, No. (%)		
ASA	80 (90)	85 (94)
P2Y12 inhibitor	66 (74)	69 (77)
Statin	82 (92)	81 (90)
β-Blocker	33 (37)	30 (33)
ACE inhibitor	34 (38)	41 (46)
NOAC	6 (7)	6 (7)
Anticoagulant	13 (15)	14 (16)
Coumarin	7 (8)	8 (9)

Abbreviations: ACE, angiotensin converting enzyme; ASA, acetylsalicylic acid; BMI, body mass index (calculated as weight in kilograms divided by square of height in meters); CABG, coronary artery bypass grafting; DBP, diastolic blood pressure; HbA_{1c}, hemoglobin A_{1c}; LDL, low-density lipoprotein; MCR, mobile cardiac rehabilitation; NOAC, novel oral anticoagulant; PCI, percutaneous coronary intervention.
SI conversion factors: To convert cholesterol to mmol/L, multiply by 0.0259; cm to feet, divide by 30; km to miles, divide by 1.6.

© 2020 Snoek JA et al. *JAMA Cardiology.*

eTable 2. Primary and Secondary Outcome Parameters at Baseline and 6- and 12-Month Follow-up

	***m*CR**			**p-value***		**Control**			**p-value***	
Parameter	**0 months**	**6 months**	**12 months**	**Δ0-6**	**Δ0-12**	**0 months**	**6 months**	**12 months**	**Δ0-6**	**Δ0-12**
CPET										
VO_{2peak} ($mL\cdot min^{-1}$)	1536 ± 495	1653 ± 520	1648 ± 519	**<0.001**	**0.001**	1631 ± 510	1641 ± 498	1653 ± 493	0.67	0.44
$VO_{2peak}\cdot kg^{-1}$ ($mL\cdot kg^{-1}min^{-1}$)	18.9 ± 5.4	20.6 ± 6.0	20.3 ± 5.7	**<0.001**	**0.003**	20.3 ± 5.7	20.5 ± 5.4	20.6 ± 5.4	0.53	0.83
Peak workload (Watt)	126 ± 54	134 ± 61	136 ± 60	**0.001**	**<0.001**	129 ± 50	131 ± 51	134 ± 52	0.29	**0.02**
HR_{max} (bpm)	127 ± 22	126 ± 20	128 ± 23	0.66	0.62	126 ± 22	126 ± 22	126 ± 22	0.85	0.68
RER	1.11 ± 0.08	1.10 ± 0.10	1.14 ± 0.11	0.11	0.06	1.10 ± 0.09	1.10 ± 0.09	1.14 ± 0.10	0.96	**0.002**
RPE (BORG)	14.6 ± 3.0	14.4 ± 2.9	14.9 ± 2.7	0.28	**0.04**	14.9 ± 2.9	15.0 ± 2.7	15.1 ± 2.7	0.74	0.62
CV risk factors										
BMI ($kg\cdot m^{-2}$)	27.6 ± 4.1	27.6 ± 3.9	27.4 ± 3.8	0.68	0.86	27.2 ± 4.2	27.4 ± 4.3	27.5 ± 4.2	0.11	**0.02**
SBP (mmHg)	134 ± 18	133 ± 20	132 ± 15	0.83	0.29	130 ± 17	133 ± 18	129 ± 17	0.13	0.31
DBP (mmHg)	78 ± 11	76 ± 12	74 ± 13	0.19	**0.01**	76 ± 12	78 ± 11	74 ± 11	**0.03**	**0.05**
Blood tests										

© 2020 Snoek JA et al. *JAMA Cardiology.*

Cholesterol (mmol·L^{-1})	4.1 ± 1.0	4.2 ± 1.0	4.1 ± 1.0	0.15	0.45	4.2 ± 0.9	4.3 ± 1.0	4.1 ± 1.0	0.23	0.94
LDL (mmol·L^{-1})	2.2 ± 0.7	2.2 ± 0.8	2.2 ± 0.8	0.37	0.51	2.3 ± 0.7	2.3 ± 0.8	2.1 ± 0.7	0.79	0.20
HDL (mmol·L^{-1})	1.3 ± 0.4	1.5 ± 0.4	1.4 ± 0.4	**<0.001**	**0.002**	1.3 ± 0.4	1.4 ± 0.5	1.4 ± 0.5	**0.002**	**0.001**
HbA1c (mmol·mol^{-1})	42.6 ± 9.4	42.7 ± 10.0	42.0 ± 8.1	0.89	0.52	40.0 ± 5.7	40.8 ± 6.0	42.0 ± 8.2	0.06	**0.004**
Questionnaires										
PHQ-9	3.3 ± 3.0	3.0 ± 3.5	3.3 ± 3.7	0.42	0.85	3.4 ± 3.2	4.0 ± 4.4	3.3 ± 3.7	0.17	0.92
SF36v2 physical	48.5 ± 6.9	50.2 ± 7.2	50.6 ± 7.2	**0.03**	**0.008**	48.1 ±8.3	48.3 ± 7.5	49.0 ± 8.2	0.81	0.26
SF36v2 mental	53.5 ± 8.0	54.0 ± 8.4	53.2 ± 8.8	0.56	0.95	53.2 ± 7.1	52.7 ± 9.1	52.5 ± 9.2	0.59	0.54
GAD-7	1.7 ± 2.6	1.8 ± 3.1	1.7 ± 2.9	0.66	1.00	1.7 ±2.7	2.4 ± 4.0	1.7 ± 3.4	0.11	0.59
MVPA (days·week^{-1})	4.2 ± 2.6		5.8 ± 1.7		**<0.001**	4.3 ± 2.5		5.2 ± 2.5		**0.008**

Data are expressed as means ± standard deviation (SD). VO_{2peak}, peak oxygen uptake; $VO_{2peak}·kg^{-1}$, peak oxygen uptake per bodyweight; Peak workload; HR_{max} maximal heart rate; RER, respiratory exchange ratio; RPE, rate of perceived exertion; HRR_{1M}, heart rate recovery in 1 minute; BMI, body mass index; SBP, systolic blood pressure; DPB, diastolic blood pressure; Cholesterol; LDL, low density lipoprotein; HDL, high density lipoprotein; HbA1c; PHQ-9, patient health questionnaire; SF36, short form; GAD 7, general anxiety disorder questionnaire; MVPA, moderate to vigorous physical activity; * p-values from paired sample t-test

© 2020 Snoek JA et al. *JAMA Cardiology.*

eTable 3. Adverse Events Within Study Period

**Event**	***m*CR (n=89)**	**Control (n=90)**	**p-value**
All-cause mortality	1 (1)	0	0.50
Cardiovascular mortality	1 (1)	0	0.50
Near sudden cardiac death	1 (1)	1 (1)	1.00
Acute coronary syndrome	4 (4)	3 (3)	0.72
Hospitalization cardiac reason	11 (12)	8 (9)	0.48
CCS	6	2	
ACS	4	2	
PM	0	2	
PCI	0	1	
Endocarditis	1	0	
Dyspnea	0	1	
**Total***	12 (13)	10 (11)	0.66

Data are expressed as number with percentages; *m*CR, mobile cardiac rehabilitation; CCS chronic coronary syndrome; ACS, acute coronary syndrome; PM, pacemaker; PCI, percutaneous coronary intervention; * Total number of individual patients with an adverse event

© 2020 Snoek JA et al. *JAMA Cardiology.*
