# Supplementary material for: Effectiveness of Home-Based Mobile Guided Cardiac Rehabilitation as Alternative Strategy for Nonparticipation in Clinic-Based Cardiac Rehabilitation Among Elderly Patients in Europe: A Randomized Clinical Trial
Source: JAMA Cardiol. 2020 Oct 28;6(4):1–6. doi: 10.1001/jamacardio.2020.5218 (PMC7593879; doi:10.1001/jamacardio.2020.5218)
Supplement: Supplement 3. — Data Sharing Statement [file jamacardiol-e205218-s003.pdf]

Data Sharing Statement

Data

Data available: Yes
Data types: Deidentified participant data
How to access data: e.p.de.kluiver@isala.nl
When available: With publication

Supporting Documents

Document types: Statistical/analytic code, Informed consent form
How to access documents: e.p.de.kluiver@isala.nl
When available: With publication

Additional Information

Who can access the data: Data (de-identified participant data) that support the findings of this study, the statistical analysis plan and informed consent forms are available from the corresponding author upon reasonable request within two years after online publication.
Types of analyses: Meta-analysis
Mechanisms of data availability: with investigator support, after approval of a proposal.
